# Green synthesis of a recyclable Ag/rGO@LS nanocomposite: utilizing *Piper chaba* stem extract for the one-pot immobilization of Ag/rGO onto luffa sponge scaffolds

**DOI:** 10.1039/d6ra04267h

**Published:** 2026-08-13

**Authors:** Prianka Saha, Md. Mahiuddin

**Affiliations:** a Chemistry Discipline, Khulna University Khulna 9208 Bangladesh mahiuddin@chem.ku.ac.bd

## Abstract

This study details the green and environmentally benign one-pot aqueous-phase synthesis of a silver nanoparticle/reduced graphene oxide immobilized luffa sponge (Ag/rGO@LS) ternary nanohybrid, utilizing *Piper chaba* stem extract. UV-vis, FTIR, Raman, and EDX confirmed the successful synthesis of the Ag/rGO binary nanocomposite. Spectra exhibited characteristic rGO (255 nm) and AgNP (407 nm) peaks. XRD validated the crystalline nature of the AgNPs, while SEM and TEM confirmed spherical particles (5–20 nm) anchored to exfoliated rGO sheets. Finally, XRD and SEM analyses verified the successful immobilization of the Ag/rGO binary system onto the LS framework and the formation of the Ag/rGO@LS ternary nanohybrid. The synthesized Ag/rGO nanocomposite demonstrated significant catalytic activity for the rapid reduction of 4-nitrophenol (4-NP) to 4-aminophenol (4-AP) in the presence of NaBH_4_. This reduction was completed in 9 min, which is substantially faster than that achieved by AgNPs (24 min) or (AgNPs and rGO) the mixture (13 min). The enhanced catalytic performance is attributed to the synergistic properties facilitating electron transport. However, the Ag/rGO nanocomposite showed poor reusability (49% of reduction after 4^th^ cycle). In contrast, the ternary Ag/rGO@LS hybrid showed excellent reusability (∼100% at 5^th^ cycle) and retained its structural integrity as observed by SEM analysis. This work supports a sustainable route for producing functional nanomaterials for environmental remediation applications.

## Abstract 2

Biogenic one-pot synthesis of Ag/rGO@LS using *Piper chaba* stem extract produced a stable, recyclable nanohybrid catalyst for 4-NP reduction, maintaining ∼100% conversion efficiency over five consecutive cycles with good structural integrity.

## Graphical abstract

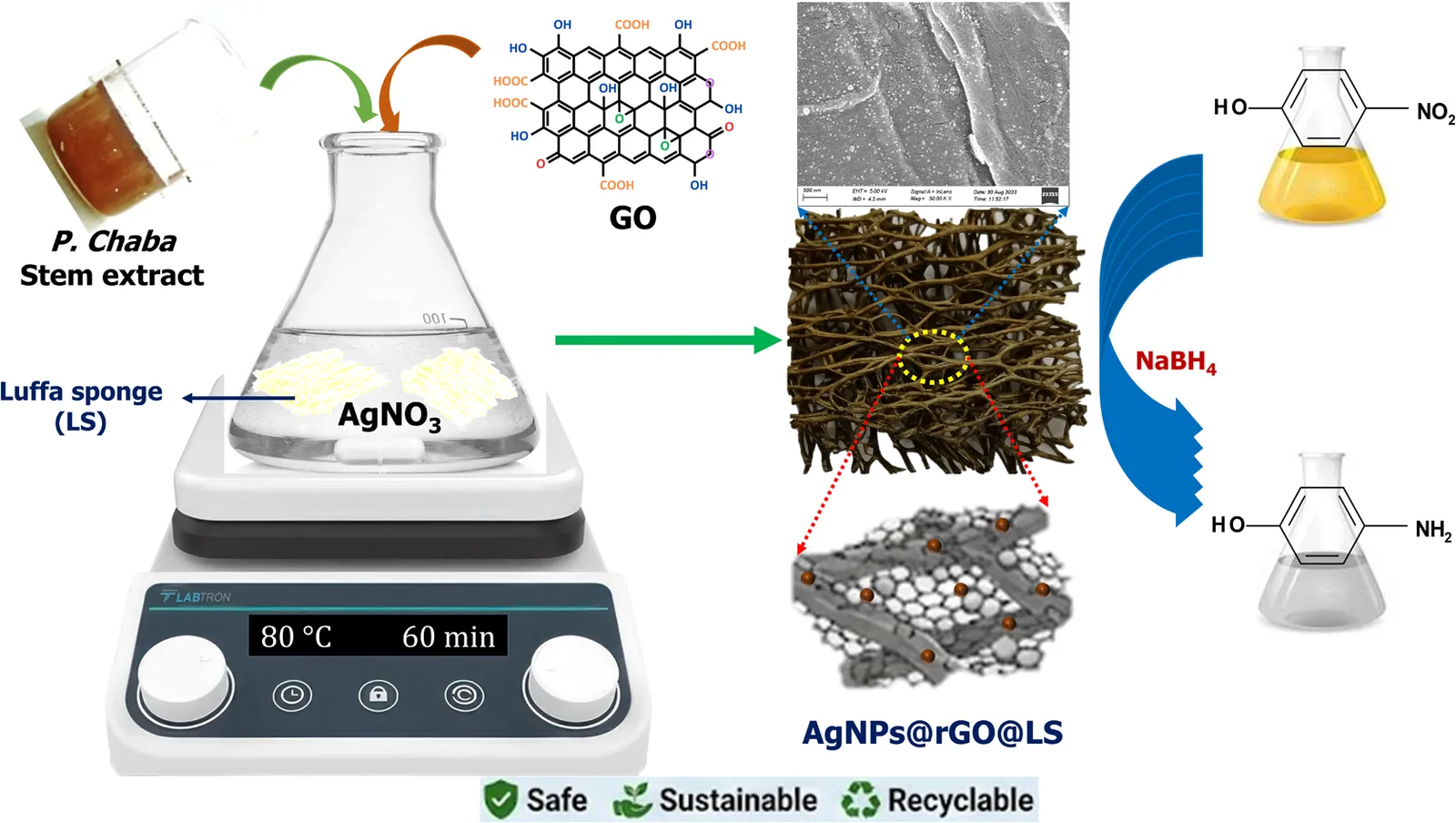

## Introduction

The twenty-first century presents critical, intertwined global challenges that demand innovative solutions from materials science: the pervasive contamination of aquatic environments by persistent organic pollutants (POPs).^1–5^ Among these contaminants, nitroaromatic compounds like 4-nitrophenol (4-NP)—byproducts of the pesticide, dye, and pharmaceutical industries—are particularly concerning due to their high solubility, chemical stability, and severe toxicological impacts on human health, including liver damage and systemic irritation.^6–9^ The catalytic reduction of 4-NP to 4-aminophenol (4-AP) has emerged as a preferred remediation strategy; not only does it mitigate toxicity, but it also generates 4-AP, a high-value intermediate for the synthesis of analgesic drugs and corrosion inhibitors.^10–13^

Nanotechnology provides a robust platform for such environmental solutions, specifically through the use of silver nanoparticles (AgNPs). Silver is favored for its potent, broad-spectrum catalytic activity and relative cost-effectiveness compared to other noble metals.^14–16^ To maximize performance, AgNPs are frequently integrated with reduced graphene oxide (rGO) to form Ag/rGO nanocomposites.^17–19^ As a support, rGO restores the π-conjugated sp^2^ carbon network, providing an ultra-high surface area and acting as an electron mediator that facilitates rapid redox reactions through synergistic electron transfer.^20–23^

Despite their high catalytic potency, Ag/rGO nanocomposites face significant practical hurdles. In aqueous suspensions, these materials are highly prone to agglomeration, which drastically reduces their active surface area and catalytic efficiency.^24,25^ Furthermore, recovering these fine particles after reaction is energy-intensive, often requiring high-speed centrifugation, and failure to do so results in secondary pollution *via* nanoparticle leaching.^20^ Additionally, the conventional synthesis of Ag/rGO nanocomposites often relies on hazardous reducing agents such as sodium borohydride (NaBH_4_) and hydrazine hydrate (N_2_H_4_).^26,27^ These chemicals are corrosive and carcinogenic, posing severe risks to biological systems and undermining the “green” objectives of environmental remediation.

While biogenic synthesis (phyto-mediated synthesis) using plant extracts has been proposed as a sustainable alternative, a significant gap remains in developing robust, one-pot protocols that yield stable, multifunctional Ag/rGO nanocomposites ready for industrial application.^28–31^ Many existing green methods lack sufficient control over nanoparticle distribution and colloidal stability. Furthermore, there is limited research on the simultaneous biogenic reduction and immobilization of these composites onto natural, 3D scaffolds to resolve the twin challenges of agglomeration and recyclability.^26,32^

This study proposes a novel, one-pot biogenic synthesis of Ag/rGO nanocomposites using the aqueous stem extract of *Piper chaba* (Chui Jhal). *P. chaba* is an abundant, edible plant in South Asia characterized by a rich phytochemical profile including polyphenols, flavonoids (*e.g.*, kusunokinin and pellitorine), and alkaloids (*e.g.*, chabamide).^33,34^ These moieties serve a dual function as potent reducing agents for Ag^+^ and GO, and as natural capping agents that ensure long-term stability *via* anionic repulsion.^35,36^ To address reusability, the composite is immobilized onto a luffa sponge (LS) (*Luffa cylindrica*) through a one-pot synthesis protocol. The LS provides a highly porous, lignocellulosic 3D micro-network that prevents nanoparticle leaching while ensuring high mass transfer and ease of mechanical recovery.^26,37,38^

The porous architecture of a LS prevents leaching through a combination of mechanical interlocking and strong intermolecular bonding. The hierarchical macropores physically confine the Ag/rGO nanocomposites within the lignocellulosic matrix, while the high surface area facilitates extensive hydrogen bonding between the hydroxyl groups of the LS cellulose and the oxygenated moieties of rGO.^39^ Furthermore, robust π–π stacking and van der Waals interactions between the rGO sheets and the LS fibers, coupled with the coordination of AgNPs to the rGO scaffold, create a stable 3D heterostructure that resists hydraulic shear and minimizes nanoparticle detachment during washing.^39,40^

The novelty of this investigation lies in the development and comprehensive performance validation of an Ag/rGO nanocomposite immobilized onto a luffa sponge, synthesized *via* a unique, single-step, and environmentally benign route utilizing *P. chaba* stem extract. By leveraging the rich phytochemical profile of *P. chaba* as a dual-action reducing and stabilizing agent, this research aims to simultaneously synthesize and anchor the Ag/rGO architecture onto the hierarchical 3D lignocellulosic scaffold of the luffa sponge. The primary objectives are to successfully produce a stable, macro-scale catalytic platform that circumvents the toxicological drawbacks of conventional chemical reduction methods and to critically evaluate its performance as a highly recyclable nanocatalyst for the conversion of 4-NP to 4-AP. Ultimately, this study seeks to establish a sustainable and scalable framework for environmental remediation by ensuring high catalytic efficiency and easy mechanical recovery over multiple cycles.

## Materials and methods

### Materials

Graphite powder was obtained from Qualikems Fine Pvt. Ltd (India), while sulfuric acid (H_2_SO_4_) was purchased from VWR International Ltd (Leicestershire, UK). Sodium nitrate (NaNO_3_), potassium permanganate (KMnO_4_), and hydrogen peroxide (H_2_O_2_, 30%) were procured from Merck (Germany). Hydrochloric acid (HCl, 37%), sodium hydroxide (NaOH), and ethanol were purchased from Loba Chemicals Ltd (India). Silver nitrate was obtained from Sigma-Aldrich (UK). 4-Nitrophenol (4-NP) and sodium borohydride (NaBH_4_) were obtained from Tokyo Chemical Industry (Tokyo, Japan). Accordingly, methylene blue (MB) and methyl orange (MO) were supplied by Sigma-Aldrich (Merck KGaA, Darmstadt, USA). All chemicals were of analytical grade and used as received without further purification. Deionized distilled water (DDW) was utilized as the solvent for all experimental procedures.

### Measurements

The physicochemical properties of the fabricated AgNPs@rGO@LS composites were systematically characterized using a suite of analytical techniques. Optical absorption profiles were acquired *via* a SHIMADZU UV-1900i spectrophotometer across a 190–1100 nm spectral window at a 1 nm resolution. Surface functional groups were identified through Fourier transform infrared (FTIR) spectroscopy (SHIMADZU IRSpirit) using the KBr pellet method, scanning from 400 to 4000 cm^−1^ at a rate of 4 cm^−1^ s^−1^. Morphological evaluations and elemental mapping were performed using a ZEISS Sigma-300 electron microscope, using an acceleration voltage of 5 kV. For FE-SEM characterization, an aqueous suspension of products was drop cast onto aluminum sheet, dried in air, and sputter coated with gold. Internal nanostructures were resolved using a JEOL TEM-2100F field emission transmission electron microscope. Crystalline phases were determined by X-ray diffraction (XRD) on a Rigaku SmartLab diffractometer (Tokyo, Japan) with Cu-Kα radiation (*λ* = 1.5406 Å) operating at 33 kV and 45 mA over a 2*θ* range of 5° to 80° with a scan rate of 1° min^−1^, while structural defects in rGO were assessed *via* Raman spectroscopy (JASCO RPM-320) using a 532 nm Ar-ion laser. Finally, thermal stability was monitored under a nitrogen atmosphere using a Seiko TG/DTA 6200 analyzer, heating from room temperature at a constant ramp of 10 °C min^−1^.

### Preparation of extract

In accordance to the previous reports,^35,36,41^ the fresh stem of *P. chaba* plant was collected from the Khulna District of Bangladesh. The collected stem was meticulously rinsed with deionized distilled water, gently chopped into small pieces using a sharp knife, and dried completely at room temperature. To obtain the *P. chaba* stem extract, approximately 20 g of the stem was placed in a 500 mL round-bottom flask holding 250 mL of DDW and boiled for 40 minutes. The extract was subsequently cooled to ambient temperature, filtered through Whatman No. 1 filter paper, centrifuged at 11 000 rpm, and kept at 4 °C for future use.

### Synthesis of Ag/rGO nanocomposites

**Scheme 1:**
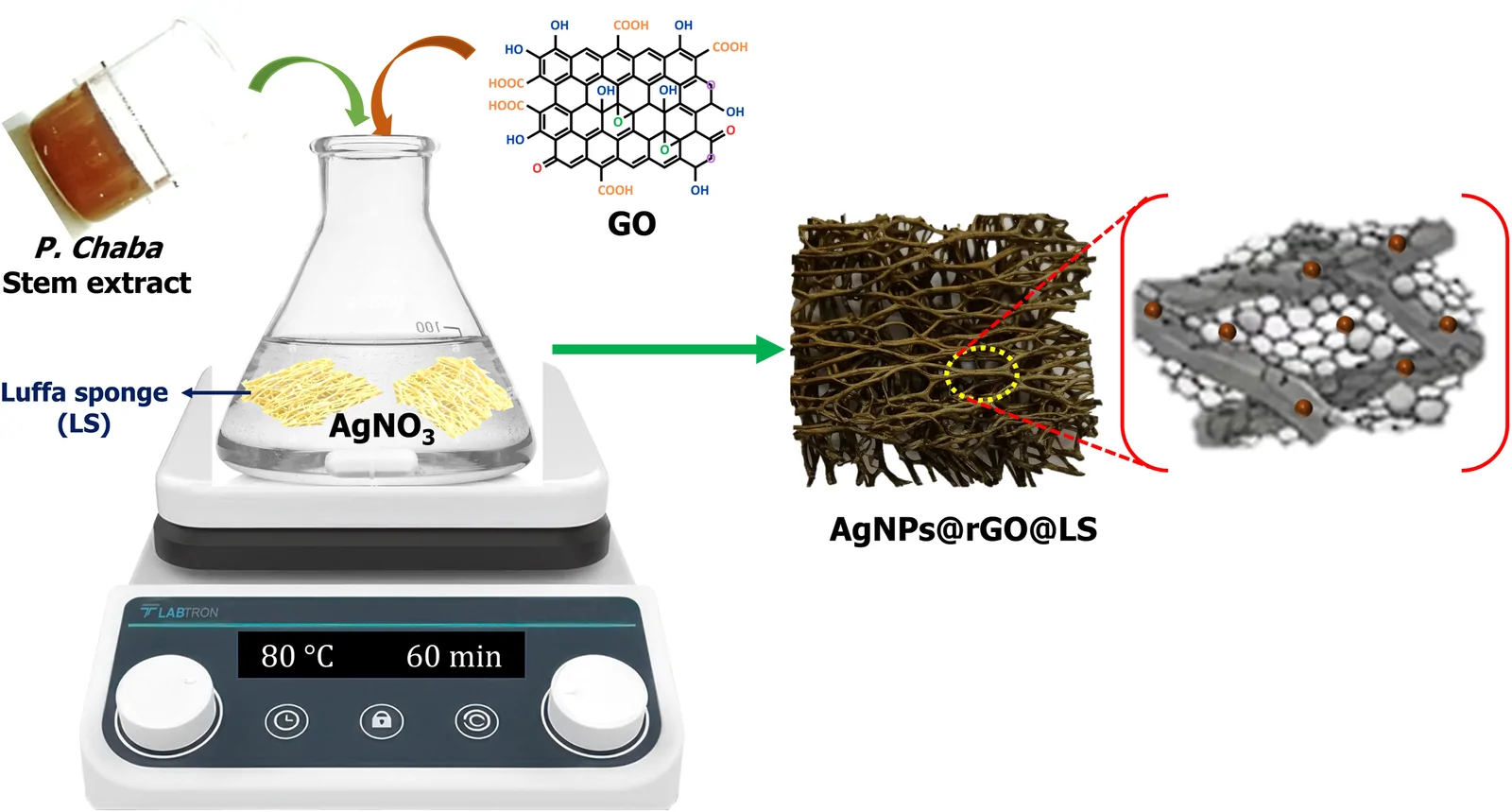
Fabrication of Ag/rGO nanocomposite on luffa sponge (LS) (Ag/rGO@LS) carried out *via* and one-pot approach using *P. chaba* stem extract as reducing and stabilizing agent.

Graphene oxide (GO) was synthesized *via* a modified Hummers' method, following the protocols established in our earlier work.^36,42,43^ The Ag/rGO nanocomposites were fabricated through a facile, one-step green reduction process utilizing *P. chaba* stem extract as the reducing and stabilizing agents. Initially, 10 mg of GO was ultrasonically dispersed in 4 mL of deionized water. To this suspension, 40 mL of AgNO_3_ solution was added at varying molar concentrations (2, 4, 6, and 8 mM). The mixture was stirred for 30 minutes to ensure homogeneous ion distribution. Subsequently, 6 mL of the *P. chaba* stem extract was introduced into the mixture. The solution pH was adjusted to 12 using 4 M NaOH. The reduction reaction was conducted in an oil bath maintained at 80 °C for 1 h. Upon completion, the reaction mixture was allowed to cool to ambient temperature. The resulting products were isolated *via* centrifugation at 11 000 rpm. The recovered solids were washed repeatedly with DDW to remove residual precursors, then dried under vacuum at 60 °C for 24 h. The samples were stored at 25 °C and labeled Ag/rGO-2, Ag/rGO-4, Ag/rGO-6, and Ag/rGO-8, reflecting the initial AgNO_3_ concentrations. Meanwhile, rGO and AgNPs were synthesized under identical experimental conditions (using 4 mM AgNO_3_ for the AgNPs). Additionally, fabrication of Ag/rGO nanocomposite on luffa sponge (LS) (Ag/rGO@LS) carried out *via* the identical one-pot approach (Scheme 1) by incorporating GO, 4 mM AgNO_3_, and treated LS into the reaction mixture. The LS sponge underwent a preliminary pre-treatment step (as describe in SI) prior to this one-pot *in situ* synthesis and immobilization process. Upon completion of the reaction, the fabricated Ag/rGO@LS was carefully retrieved using forceps. The modified sponge was washed thoroughly with DDW, then dried under vacuum at 60 °C for 24 h and stored in a desiccator prior to characterization and catalytic testing.

### Catalytic activity

The catalytic efficiency of the Ag/rGO nanocomposite was evaluated *via* the aqueous-phase reduction of 4-NP at a constant temperature of 25 °C. In a typical procedure, 500 µL of the colloidal Ag/rGO dispersion (0.67 mg mL^−1^) was introduced into 20 mL of a 20 ppm 4-NP solution. The reaction was initiated by the addition of 15 mL of a freshly prepared aqueous solution of NaBH_4_ (0.02 M). The progress of the reduction was monitored in real-time using UV-vis spectrophotometry, with absorption spectra recorded across a wavelength range of 250 to 800 nm. For comparative purposes, control experiments were performed under identical conditions using rGO, AgNPs, a physical mixture of AgNPs and rGO synthesized using *P. chaba* stem extract, and a blank sample (without catalyst). To assess the stability and reusability of the Ag/rGO nanocatalyst, the material was recovered post-reaction *via* centrifugation, washed thoroughly with DDW, and reintroduced into fresh 4-NP cycles. Throughout these cycles, the initial concentration of 4-NP, the volume of the reactants, and the reaction duration were kept constant. The catalytic reduction of 4-nitrophenol was monitored using real-time UV-vis spectroscopy, and representative spectral profiles are presented to illustrate kinetic absorption changes.

Furthermore, the catalytic performance was tested against organic dyes, specifically MO and MB. These trials followed the aforementioned protocol, with appropriate adjustments made to the initial dye concentrations and the volume of the NaBH_4_ reductant to ensure optimal degradation conditions. For MB, NaBH_4_ (0.02 M, 10 mL) and MB (10 ppm, 25 mL) and for MO, NaBH_4_ (0.02 M, 5 mL) and MO (15 ppm, 30 mL).

The catalytic performance of the Ag/rGO@LS composite was evaluated using the 4-NP reduction model described previously. To ensure a valid comparison, the quantity of the LS-supported catalyst was calibrated to provide a concentration of active material equivalent to that used in the Ag/rGO. Following each catalytic cycle, the modified sponge was retrieved from the reaction medium using forceps, washed thoroughly with DDW, and reintroduced into fresh 4-NP cycles. This process was repeated for several consecutive cycles to determine the long-term stability and reusability of the supported catalyst. Catalytic reusability was evaluated over five consecutive operational cycles using a single immobilized substrate matrix to assess cumulative recovery and stability.

## Results and discussion

**Fig. 1:**
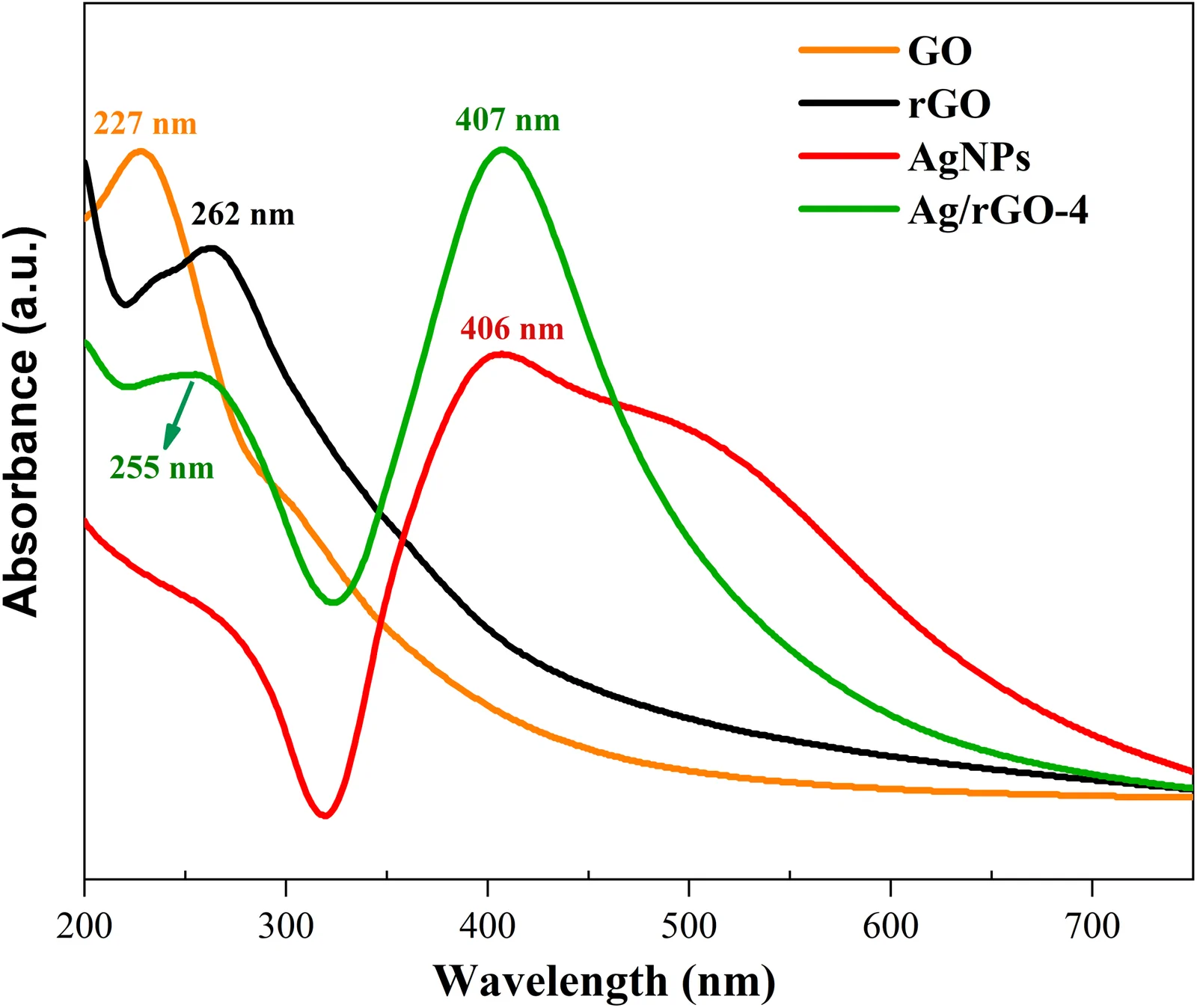
UV-vis spectra of GO, rGO, AgNPs, and Ag/rGO nanocomposite synthesized using *P*. *chaba* stem extract.

The UV-vis spectrum of GO is characterized by a strong absorption peak at 227 nm (attributed to π–π* transitions) and a shoulder at 288 nm (Fig. 1), originating from the n–π* transitions of residual oxygen functional groups (C=O).^36,42^ In the final Ag/rGO nanocomposite spectrum, the main GO peak shifts hypsochromically to 255 nm, which, along with the virtual disappearance of the 288 nm shoulder, confirms the effective deoxygenation of GO and the successful restoration of the conjugated C=C network—a hallmark of rGO.^36^ Simultaneously, the Ag/rGO nanocomposite spectrum exhibits a strong Surface Plasmon Resonance (SPR) band centered at 407 nm (Fig. 1). This signature absorption peak is definitive evidence for the formation of stable AgNPs, resulting from the reduction of Ag^+^ ions by the phytochemicals present in the *P*. *chaba* stem extract.^35^ The co-existence of the shifted rGO absorbance peak (255 nm) and the strong Ag SPR band (407 nm) provides conclusive spectroscopic confirmation of the successful, simultaneous, one-pot phyto-mediated synthesis of the stable Ag/rGO nanocomposite structure.^17,30,31,44^

**Fig. 2:**
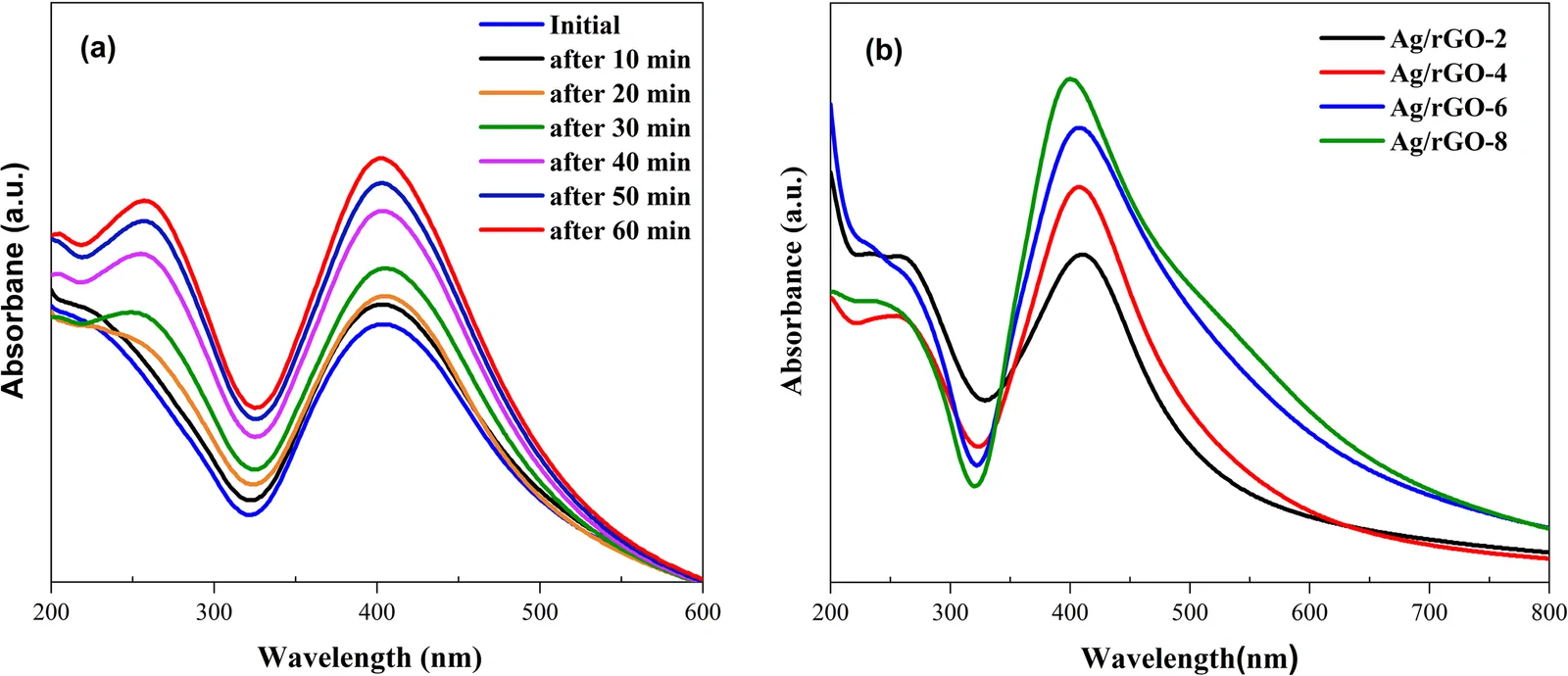
Effect of (a) reaction time and (b) initial concentration of AgNO_3_ on the behavior of Ag/rGO nanocomposites.

The UV-vis spectra were utilized for real-time monitoring of the Ag/rGO nanocomposites formation kinetics over a 60 min period. At the initial reaction phase, only the SPR peak characteristic of AgNPs (407 nm) was detectable (Fig. 2a), suggesting that the reduction of Ag^+^ ions by the *P. chaba* stem extract is kinetically favored in the early stages. The subsequent appearance of the rGO characteristic peak (255 nm) at the 20 min mark signal the initiation of GO deoxygenation. Crucially, as the reaction proceeded in 10 min intervals up to 60 min, both the rGO peak and the Ag SPR band exhibited simultaneous increases in intensity and enhanced sharpness. The growing intensity of the Ag SPR peak confirms higher nanoparticle concentration and deposition, while the increased definition of both peaks suggests improved reduction efficiency, better crystallinity, and decreased polydispersity over time, indicating a progressive stabilization of the Ag/rGO hybrid nanostructures.^5,45^

To evaluate the effect of precursor concentration on nanocomposite architecture, Ag/rGO nanocomposites were synthesized using 2, 4, 6, and 8 mM AgNO_3_. UV-vis spectra (Fig. 2b) showed that increasing AgNO_3_ concentration directly correlates with the intensity of the SPR band at 407 nm, confirming higher AgNPs loading. Beyond 4 mM, significant broadening of the SPR peak indicates increased particle size and polydispersity,^5,45–47^ while the diminishing rGO peak intensity reflects a decreased rGO-to-Ag ratio. Consequently, 4 mM AgNO_3_ was identified as the optimal concentration for maintaining high AgNPs density with controlled morphology.

The optical band gap energy was estimated from UV-vis results using the following Tauc's expression [eqn (1)]:^48^

1 (*α*ℏ*ν*)^1/*γ*^ = *B*(ℏ*ν* − *E*_g_)

where ℏ is the Planck constant, *ν* is the photon's frequency, *E*_g_ is the band gap energy, and *B* is a constant. The *γ* factor depends on the nature of the electron transition and is equal to 1/2 or 2 for the direct and indirect transition band gaps, respectively.

The optical band gap (*E*_g_) was determined by extrapolating the linear portion of Tauc plots ((*α*ℏ*ν*)^2^*vs.* ℏ*ν*) (SI, Fig. S1). Pristine GO exhibited a gap of 4.27 eV, which decreased to 3.42 eV upon reduction by *P. chaba* extract, signifying the restoration of the sp^2^ hybridized framework. Integration of AgNPs further narrowed the band gap to a range of 2.53–2.40 eV, correlating with increased metal loading. This systematic reduction suggests that AgNPs act as p-type dopants, enhancing electronic conductivity through strong interfacial interactions. These interactions are likely mediated by phytochemical residues *via* π–π stacking and electrostatic bonding, ensuring structural stability. Such electronic modifications facilitate efficient charge carrier mobility, ultimately optimizing the composite's catalytic performance.^17,49,50^

**Fig. 3:**
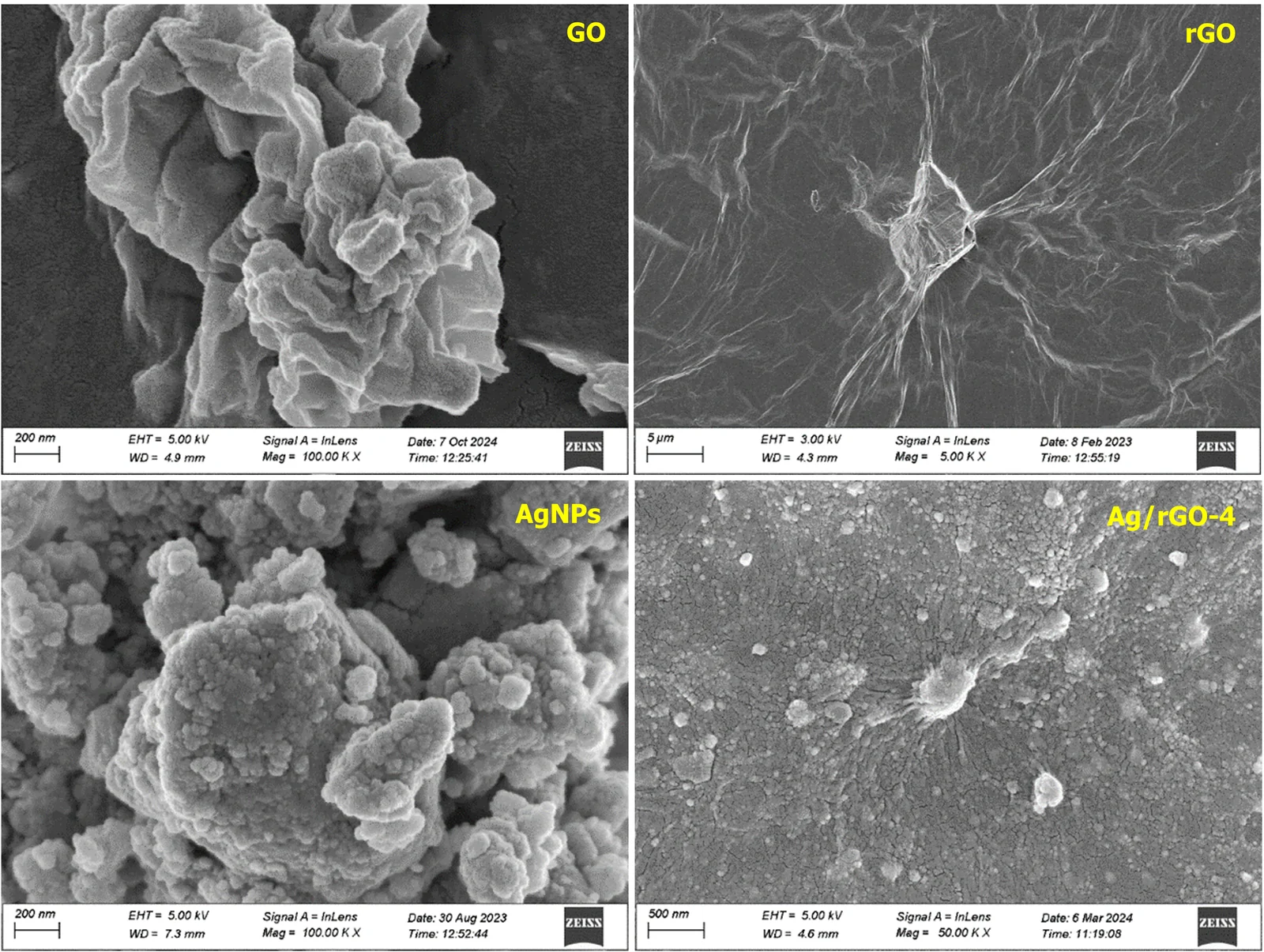
SEM images GO, rGO, AgNPs, and Ag/rGO (AgNPs@rGO-4) nanocomposite synthesized using *P. chaba* stem extract.

The SEM images of resultant materials were shown in Fig. 3. The GO precursor displayed characteristic morphology, consisting of dense, stacked, and aggregated layered sheets. Subsequent reduction yielded rGO, which showed a distinct transformation into exfoliated layers with a negligible bulk graphitic content.^36^ The biosynthesized AgNPs were visualized as aggregated, nano-sized spherical particles. Most importantly, the final Ag/rGO nanocomposite revealed successful decoration, exhibiting highly exfoliated rGO sheets hosting dispersed AgNPs of diverse sizes. This morphology confirms the effective role of the *P. chaba* stem extract in both reducing the GO^36^ and Ag^+^ ions and stabilizing the AgNPs^35^ as well as Ag/rGO nanocomposite, resulting in a well-integrated, high-surface-area composite structure ready for applications.

SEM analysis confirmed that AgNPs are supported on exfoliated rGO sheets irrespective of AgNO_3_ concentration, whereas the morphology dictated by AgNO_3_ concentration (2–8 mM) (SI, Fig. S2). Consistent with UV-vis results, higher precursor concentrations increased AgNPs size and density relative to the rGO matrix. This trend suggests that elevated Ag^+^ levels promote particle growth and aggregation over initial nucleation. The 4 mM concentration proved optimal, yielding superior dispersion and a balanced AgNPs:rGO ratio. Thus, precursor concentration acts as a critical parameter for tuning nucleation and growth kinetics during *P. chaba*-mediated synthesis to achieve precise structural control.

**Fig. 4:**
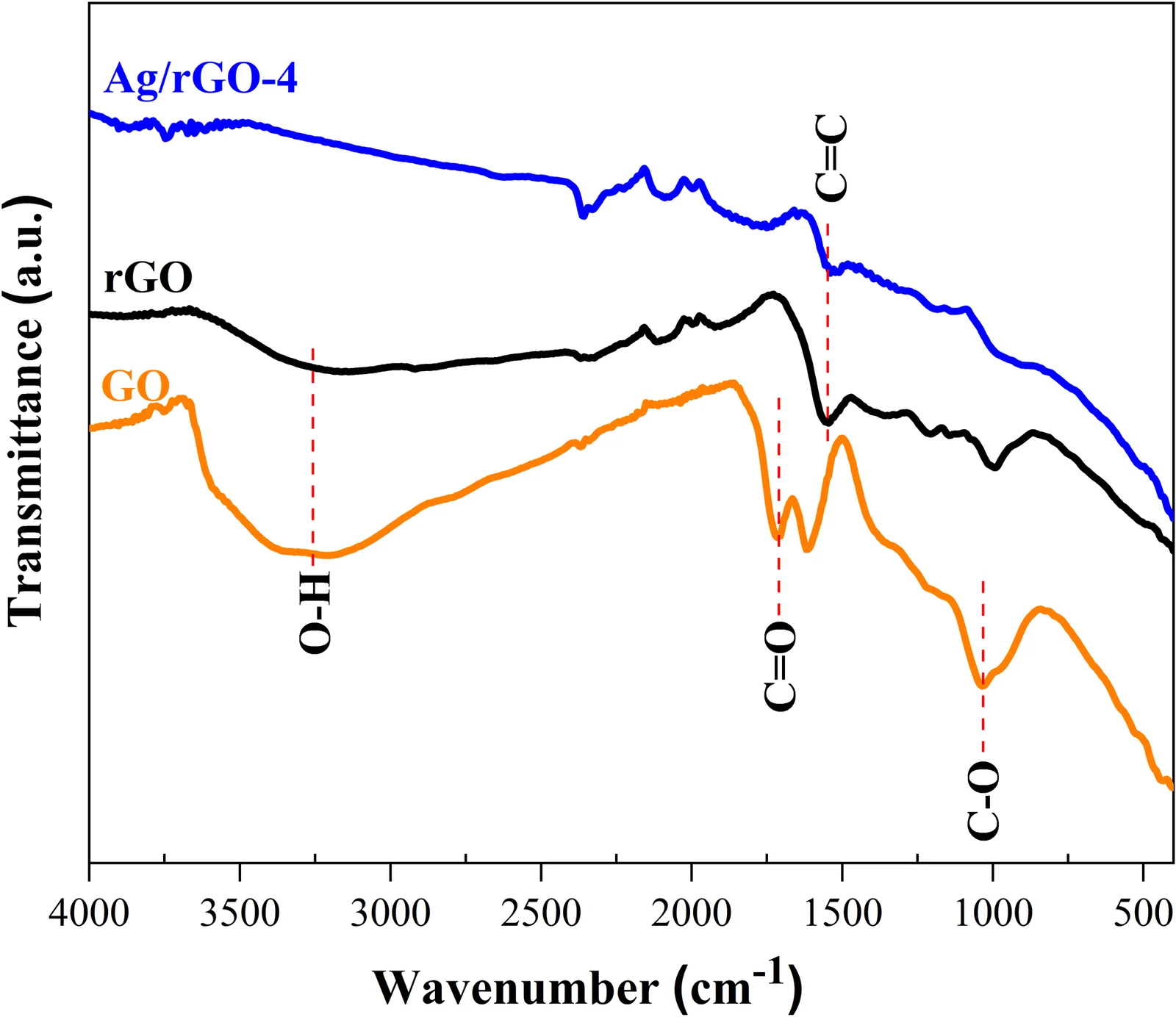
FTIR spectra of GO, rGO, and Ag/rGO (AgNPs@rGO-4) nanocomposites synthesized using *P. chaba* stem extract.

FTIR spectroscopy was employed to confirm the dual-functional role of the *P. chaba* stem extract as both a reducing and stabilizing agent. The GO spectrum (Fig. 4a) displayed characteristic signals from oxygen functionalities, including O–H stretching (3300–3400 cm^−1^), C=O stretching of carboxyl groups (1720 cm^−1^), and C–O stretching of epoxy and alkoxy groups (1220 cm^−1^ and 1050 cm^−1^). Following the phytosynthesis, the spectra of both rGO and the optimized AgNPs@rGO-4 nanocomposite showed a profound reduction or complete disappearance of these oxygen-related bands, confirming the effective deoxygenation of GO and its reduction to rGO (Fig. 4). The C=C stretching vibration, indicative of the restored graphitic framework, shifted from 1620 cm^−1^ in GO to 1545 cm^−1^ in rGO^36^ and further to 1535 cm^−1^ in Ag/rGO (AgNPs@rGO-4) nanocomposite (Fig. 4). This shift in the C=C peak in the composite suggests strong chemical or electronic coupling between the AgNPs and the rGO matrix. The systematic attenuation of residual oxygen-functional group peaks and the diminished intensity of rGO characteristic signals at higher AgNO_3_ concentrations (SI, Fig. S3a) suggest that increased AgNPs loading and subsequent aggregation effectively shield the rGO framework. Simultaneously, the reduction efficiency of GO appears positively correlated with Ag^+^ concentration, suggesting that Ag^+^ ions facilitate a synergistic reduction process. This indicates that while higher metal loading enhances the reduction of the support, the resulting dense AgNPs coverage physically suppresses the spectroscopic visibility of rGO functionalities.^51–54^

**Fig. 5:**
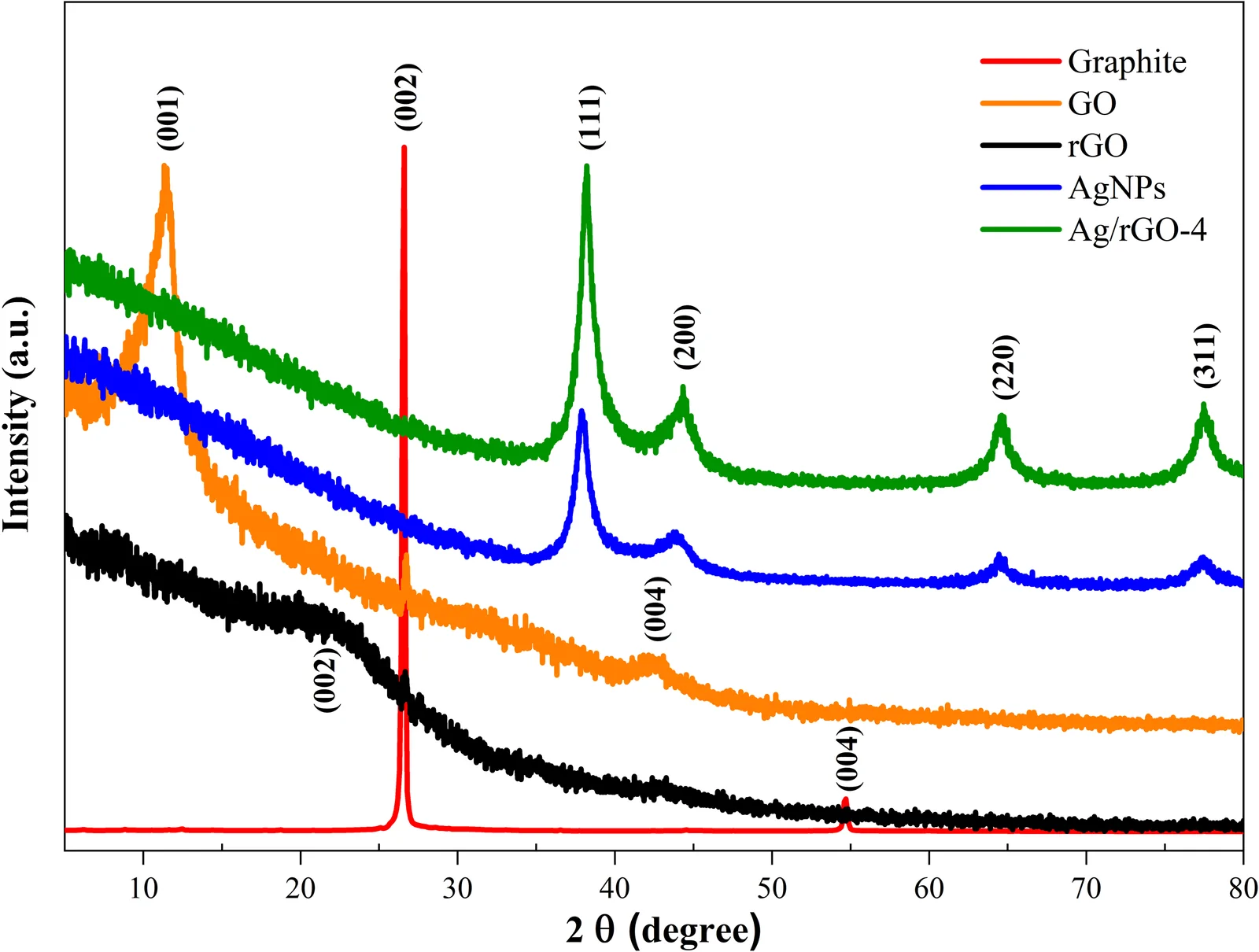
XRD patterns of graphite, GO, rGO, and Ag/rGO (AgNPs@rGO-4) nanocomposite synthesized using *P*. *chaba* stem extract.

The initial graphite showed a sharp characteristic (002) peak at 2*θ* = 26.6° (*d* = 3.33 Å), which shifted significantly in GO to a major peak at 11.5° (*d* = 7.77 Å) (Fig. 5a). This substantial increase in interplanar spacing (*d*) is characteristic of GO, indicating successful oxidation and the incorporation of oxygen-containing functional groups between the carbon layers.^55^ Reduction *via P. chaba* extract yields rGO, evidenced by the peak shifting to 22.01° (*d* = 3.55 Å). This broadening and shift toward the original graphite position signifies the removal of oxygen groups and partial restoration of the π-conjugated system.^36^ The XRD patterns for the Ag/rGOnanocomposites clearly demonstrates the presence of metallic silver. Four distinct peaks at 38.1°, 44.3°, 64.5°, and 77.5° correspond to the (111), (200), (220), and (311) planes, respectively of face-centered cubic (FCC) Ag^0^ (Fig. 5) (JCPDS card no. 04-0783).^47,56^ The absence of the 11.5° GO peak confirms comprehensive reduction. No additional reflection besides that of silver was observed, which suggests that the Ag/rGO nanocomposite lattice was unaffected by other entities.^30^ Furthermore, the progressive increase in Ag peak intensity observed with rising AgNO_3_ concentrations (2–8 mM) (SI, Fig. S3b) confirms a higher degree of crystallinity and increased Ag^(0)^ loading within the Ag/rGO nanocomposite. Calculations using the Scherrer equation indicate an expansion in average crystallite size from 7.1 nm to 9.0 nm. This concentration-dependent growth suggests that elevated precursor availability accelerates both nucleation and grain growth, ultimately surpassing the stabilizing or capping capacity of the *P. chaba* extract, leading to larger crystalline domains.

**Fig. 6:**
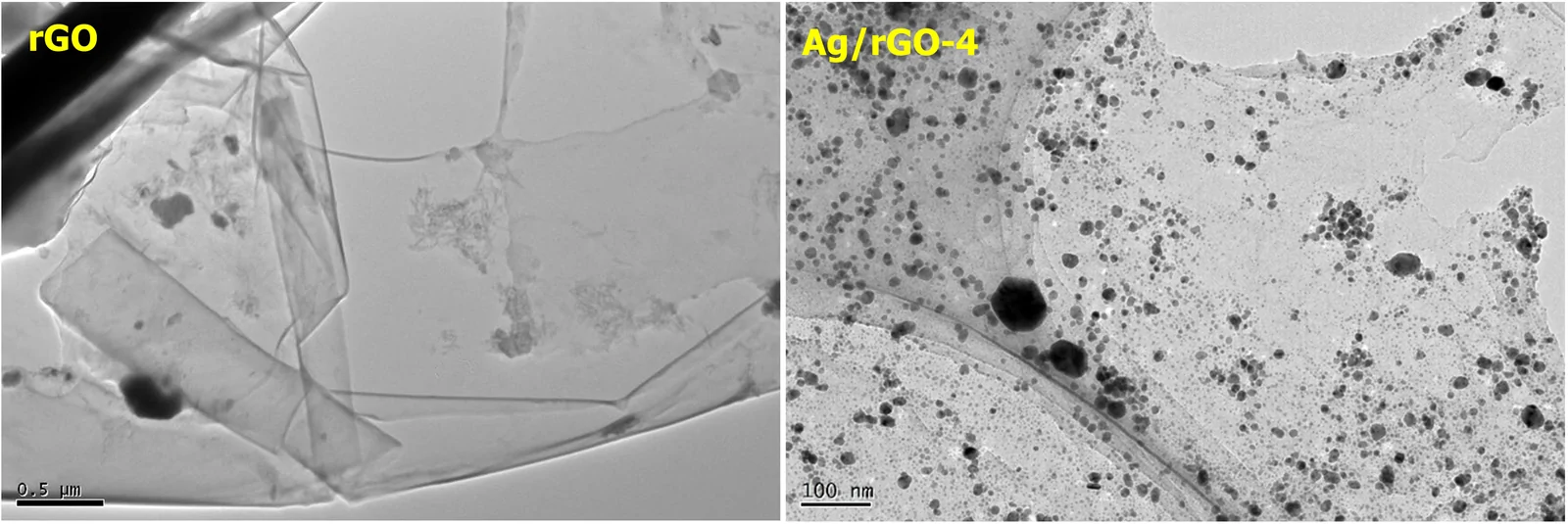
TEM images of rGO and Ag/rGO (AgNPs@rGO-4) nanocomposite synthesized using *P. chaba* stem extract.

TEM was utilized to provide a detailed view of the material microstructures as shown in Fig. 6, complementing the SEM results. The TEM image of the pristine rGO confirmed the highly successful exfoliation, displaying transparent, few-layered sheets with negligible residual bulk graphite content.^36^ In the Ag/rGO nanocomposite, TEM revealed a highly intimate association between the rGO and the AgNPs. The rGO sheets were clearly visible as exfoliated layers hosting distinct, almost uniformly sized AgNPs having the size of 5–20 nm distributed across the surface. This clear visualization of the intimate interface and the effective dispersion of the AgNPs confirms the dual reducing and stabilizing action of the *P. chaba* extract, demonstrating a high-quality, biogenic composite structure.

**Fig. 7:**
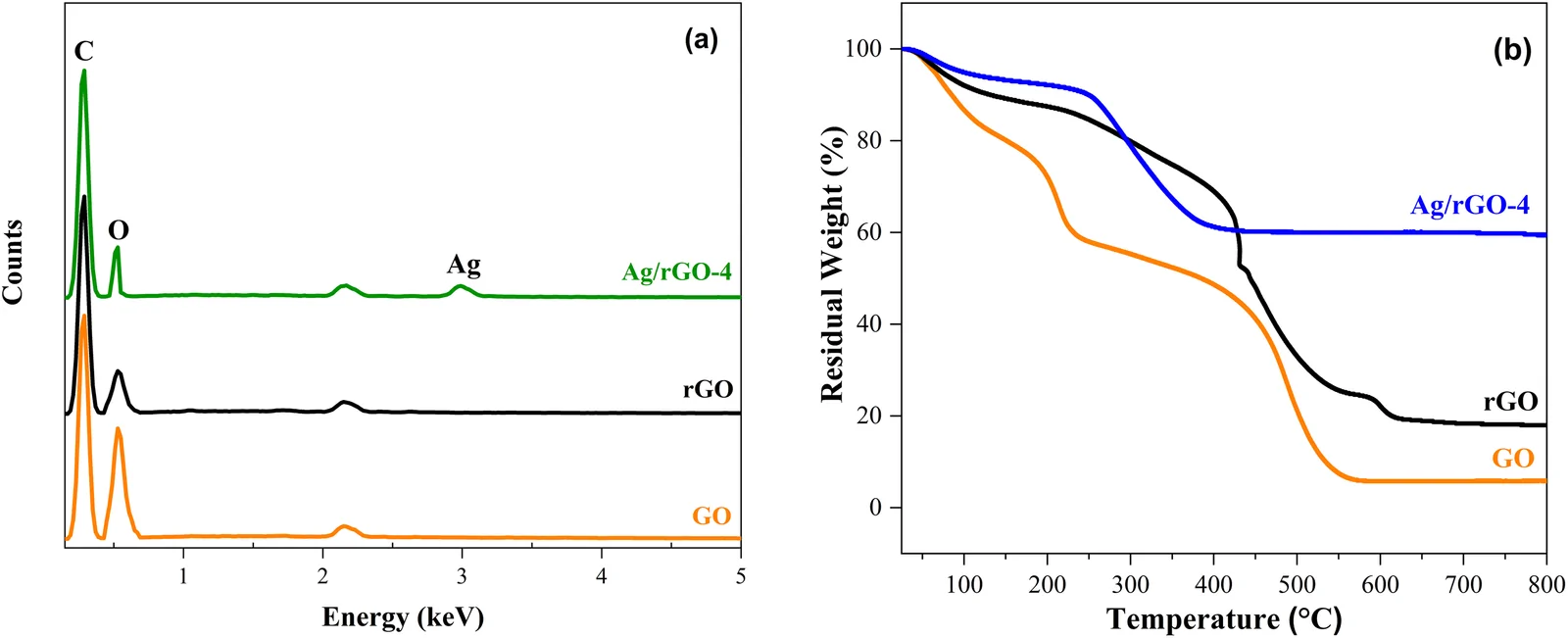
(a) EDX spectra and (b) TGA curves of GO, rGO, and Ag/rGO (AgNPs@rGO-4) nanocomposites synthesized using *P. chaba* stem extract.

EDX was used to verify the elemental composition and the reduction efficiency. The EDX spectra of GO and rGO showed strong signals for carbon (C) and oxygen (O) (Fig. 7a). A significant decrease in the oxygen signal intensity in rGO compared to GO confirmed the successful removal of oxygen-containing functional groups during the reduction process.^36^ The EDX spectrum of the Ag/rGO nanocomposite (Fig. 7a) exhibited characteristic peaks for C, O, and relatively intense signal for Ag. The oxygen content remained comparably low to rGO, indicating that the Ag decoration did not reintroduce substantial oxidation, while the presence of Ag confirms the successful biosynthesis of the AgNPs within the composite.^51,57^

TGA was employed to quantify the thermal stability and ascertain the material composition, which directly corroborates the EDX findings regarding reduction and content. The GO curve (Fig. 7b) exhibited significant weight loss below 200 °C due to the degradation of unstable oxygen-containing groups and at the temperature range of 430–520 °C due to remaining oxygen functionalities (confirming EDX's high O content). The rGO showed greatly enhanced stability specially below 200 °C, with lower overall mass loss, validating the successful reduction (EDX's low O signal).^36^ Most critically, the Ag/rGO nanocomposite demonstrated the highest thermal stability, leaving a substantial residual mass of ∼40% at 800 °C (Fig. 7b), consistent with reported silver–graphene composites.^56,58,59^ This residual weight represents the combined inorganic (Ag) and carbonaceous (rGO) components, with the large percentage definitively confirming the high content of the non-volatile AgNPs successfully incorporated into the composite.

**Fig. 8:**
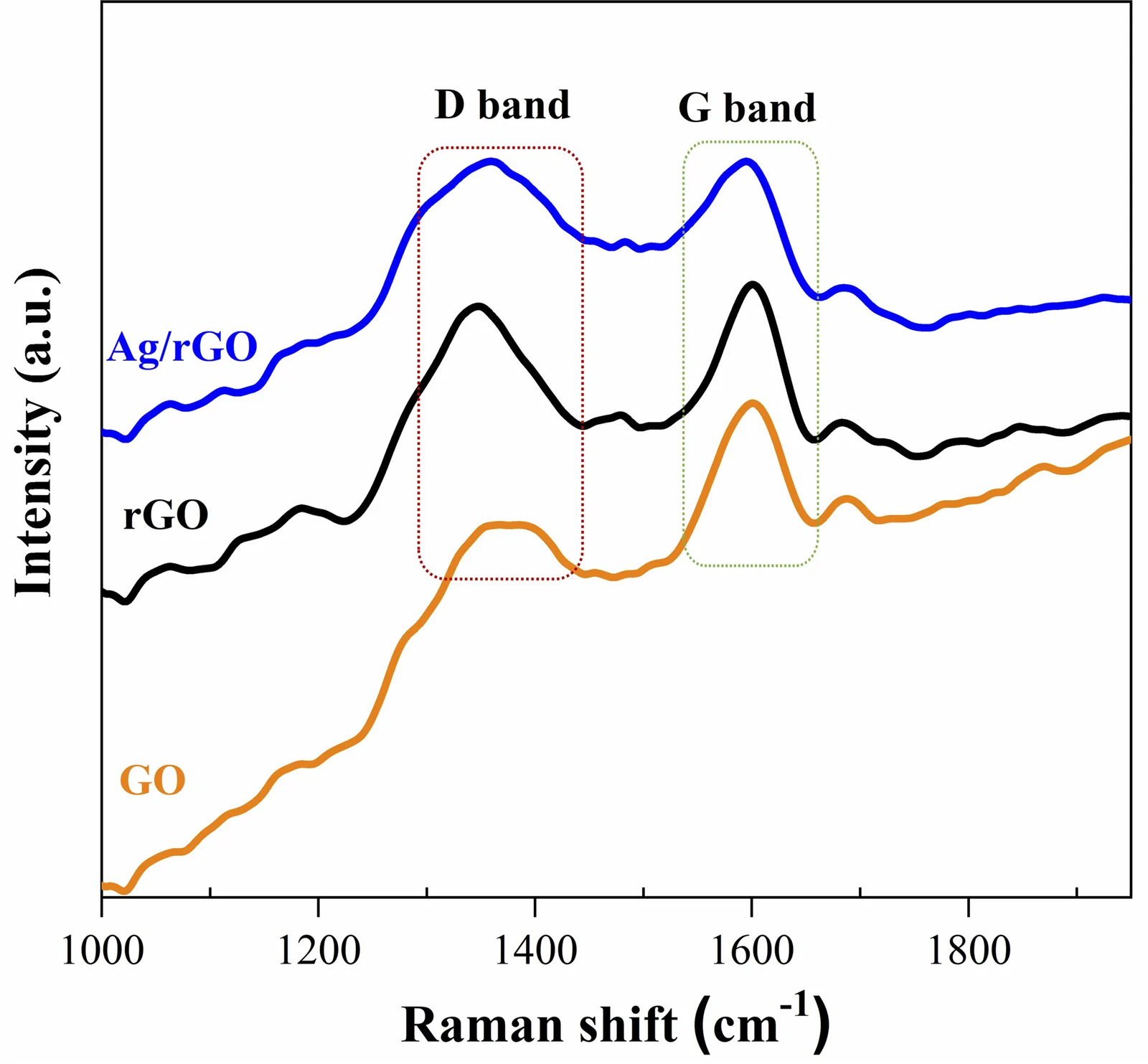
Raman spectra of GO, rGO, and Ag/rGO (AgNPs@rGO-4) nanocomposites synthesized using *P*. *chaba* stem extract.

Raman spectroscopy was conducted to evaluate the structural disorder and defect density during the transformation of GO to Ag/rGO (AgNPs@rGO). All samples exhibited the characteristic D band (disorder/defects) and G band (sp^2^ graphitic structure) (Fig. 8). For GO, the D and G bands appeared at 1372 cm^−1^ and 1600 cm^−1^, respectively, yielding an intensity ratio (*I*_D_/*I*_G_) of 0.96.^56,60^ Upon reduction to rGO, the *I*_D_/*I*_G_ ratio increased to 0.99 (D band at 1347 cm^−1^), indicating the restoration of the graphitic sp^2^ domains following the removal of oxygen functionalities, but also suggesting a simultaneous creation of smaller sp^2^ regions and increased structural defects.^36^ The final Ag/rGO nanocomposite showed a further increase in the *I*_D_/*I*_G_ ratio to 1.0 (D band at 1359 cm^−1^, G band at 1595 cm^−1^). This final increase is characteristic of defect induction due to the anchoring of AgNPs onto the rGO surface and further reduction, which disrupts the local carbon lattice and confirms the successful immobilization of the AgNPs, which is commonly found in literature.^30,61^ It should also be noted that the presence of metallic nanoparticles could potentially enhance the Raman signal through surface-enhanced Raman scattering (SERS), contributing to the increased D band intensity.

### Plausible mechanism of the formation of Ag/rGO nanocomposite

The mechanism for the *in situ* synthesis of the Ag/rGO nanocomposite begins with the mixing of highly dispersed GO sheets and silver nitrate in an aqueous medium. At alkaline condition (pH ∼ 12), the negatively charged oxygen-containing functional groups (carbonyl, hydroxyl, and epoxide) on the GO surface act as electrostatic docking and nucleation sites for the positive Ag^+^. Following the initial adsorption of Ag^+^ onto the GO support, the crucial step involves the *P. chaba* stem extract. The hydroxyl groups within the extract's polyphenols simultaneously reduce the adsorbed Ag^+^ ions to Ag^(0)^ and the GO to exfoliated rGO. Furthermore, the oxidized form of phytochemicals and the remaining bioactive compounds in the extract serve to stabilize the newly formed AgNPs against aggregation while anchoring them directly to the rGO sheets. This integrated, one-pot methodology successfully yields AgNPs decorated onto exfoliated rGO layers.^62,63^

## Application

### Catalytic activity of Ag/rGO nanocomposite

**Fig. 9:**
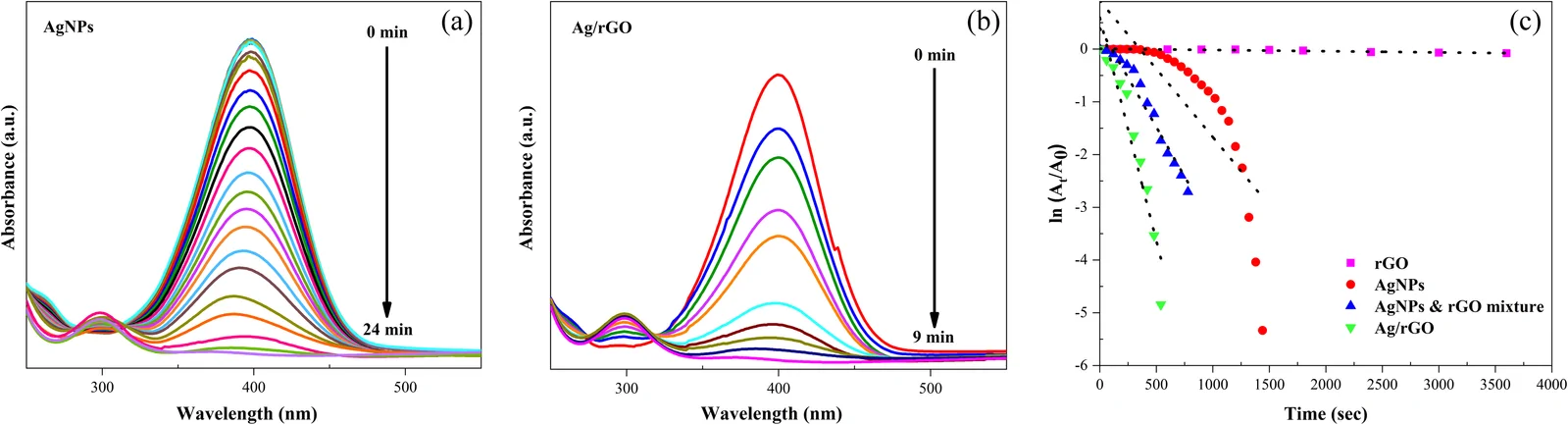
Time-dependence UV-vis spectra for the reduction of 4-NP to 4-AP by NaBH_4_ in presence of *P. chaba* stem extract-mediated (a) AgNPs and (b) Ag/rGO(AgNPs@rGO-4) as catalyst; (c) plot of ln(*A*/*A*_0_) against time.

The catalytic performance of the synthesized materials was evaluated using the reduction of 4-NP to 4-AP by NaBH_4_, a widely recognized model reaction. The reaction was conducted at ambient condition and monitored *via* UV-vis spectroscopy by tracking the disappearance of the 4-nitrophenolate ion absorption peak at 400 nm and concurrently appearance of the 4-AP peak at 300 nm. As expected, the pristine rGO showed no discernible catalytic activity, with the UV-vis spectra (SI, Fig. S4a) and solution color remaining unchanged even after 60 min, confirming that rGO primarily functions as an inert, high-surface-area support. Conversely, the AgNPs synthesized with the *P*. *chaba* extract showed activity, completing the reduction in 24 min (Fig. 9a). The Ag/rGO nanocomposite exhibited superior performance, achieving complete reduction of 4-NP (indicated by color change and total peak diminution) in a significantly shorter time of 9 min (Fig. 9b), in contrast the physical mixture of AgNPs and rGO completing the reduction in 13 min (SI, Fig. S4b), demonstrating a marked synergistic effect between the AgNPs and the rGO support in the synthesized Ag/rGO nanocomposite.^12,64,65^

Additionally, the Ag/rGO nanocatalyst demonstrated high efficiency in degrading organic dyes, with the cationic MB completely reduced in 9 min (SI, Fig. S5a), significantly faster than the anionic MO, which required 13 min (SI, Fig. S5b). This disparity is attributed to favorable electrostatic interactions: the residual negative charge on the rGO surface preferentially attracts the positive MB molecules. This enhanced localized adsorption effectively increases the concentration of MB at the AgNPs active sites, accelerating the electron transfer and subsequent degradation kinetics compared to the repulsive forces experienced by the negative MO dye.^66^

The reaction kinetics were found to follow a pseudo-first-order model, assuming the concentration of the reductant (NaBH_4_) is in large excess.^12,64,65^ The ln*A*/*A*_0_*vs.* time plots are shown in Fig. 9c and obtained kinetics parameters are in Table 1. The promising performance of the Ag/rGO nanocomposite can be attributed to the unique structure established during the *in situ* synthesis. The exfoliated rGO sheets prevent the aggregation of the AgNPs and provide an extensive surface area for reactant adsorption. Furthermore, the rGO acts as an electron relay medium, facilitating rapid electron transfer from the BH_4_^−^ ions to the AgNPs and subsequently to the 4-NP molecules, which significantly lowers the activation energy barrier for the reduction process.

**Table 1:** Results of the catalytic reduction of 4-NP to 4-AP by NaBH_4_ in the presence of Ag/rGO nanocomposite as catalyst

Catalyst	Time (min)	*R* ^2^	Reaction rate (s^−1^)	Reaction rate (s^−1^ g^−1^)
rGO	60	0.9398	0.00002	0.5970
AgNPs	24	0.6659	0.0026	77.6119
AgNPs & rGO mixture	13	0.9527	0.0037	110.4478
AgNPs@rGO	9	0.9171	0.0085	253.7313

The catalytic efficacy of the Ag/rGO heterostructure is exemplified by the reductive transformation of 4-NP to 4-AP. Consistent with established literature, the reaction kinetics are governed by the Langmuir–Hinshelwood (L–H) mechanism. According to the L–H model, the reaction proceeds on the AgNPs surface, which acts as a miniature electron reservoir. First, the 4-nitrophenolate and BH_4_^−^ ions are adsorbed onto the highly accessible surface of the AgNPs decorated on the rGO. The electrons transfer to AgNPs/rGO from BH_4_^−^ ions (hydrogen source and the electron donor), the active hydrogen species are formed, and then the active hydrogen species reduce 4-NP into 4-AP (SI, Fig. S6).^23,67^ The rGO sheet enhances this electron flow, allowing the rapid transfer of electrons across the catalyst surface to reduce the adsorbed nitrophenolate into the final product, 4-AP. The 4-AP product then desorbs, freeing the active sites for subsequent cycles.^12,65^

### Recyclability and stability study of Ag/rGO nanocomposite

The operational stability of the Ag/rGO nanocatalyst was assessed over four consecutive cycles. The catalyst maintained 99% conversion in the 1st cycle, demonstrating excellent initial efficiency. However, efficiency showed a measurable decrease in subsequent cycles, dropping to 81% in the 2nd cycle, 64% in the 3rd cycle, and 49% in the 4th cycle (SI, Fig. S7a). A noticeable decay in catalytic performance was observed for the unsupported Ag/rGO nanocomposite upon repeated usage. Post-reaction SEM analysis confirmed that this activity loss was primarily driven by significant aggregation of the Ag/rGO nanostructures after the second cycle (SI, Fig. S7b), which severely reduced the exposed surface area of the active Ag nanoparticles and restricted reactant access to the catalytic sites. Conversely, the post-reaction EDX spectrum (SI, Fig. S7c) revealed strong elemental signals for carbon and silver comparable to those of the fresh catalyst. This confirms that the decline in activity stems predominantly from physical morphological changes rather than severe chemical degradation or substantial Ag leaching, although minor contributions from active site poisoning *via* 4-AP adsorption and trace leaching cannot be entirely excluded. To overcome these inherent limitations, particularly nanoparticle agglomeration and the technical challenges associated with recovering powder catalysts, the Ag/rGO nanocomposite was immobilized *in situ* onto the 3D cellulosic matrix of a luffa sponge (Ag/rGO@LS). The porous sponge framework effectively suppresses particle agglomeration, enhances structural stability, and enables seamless catalyst recovery without significant loss of active sites.

### Fabrication of AgNPs@rGO@LS

**Fig. 10:**
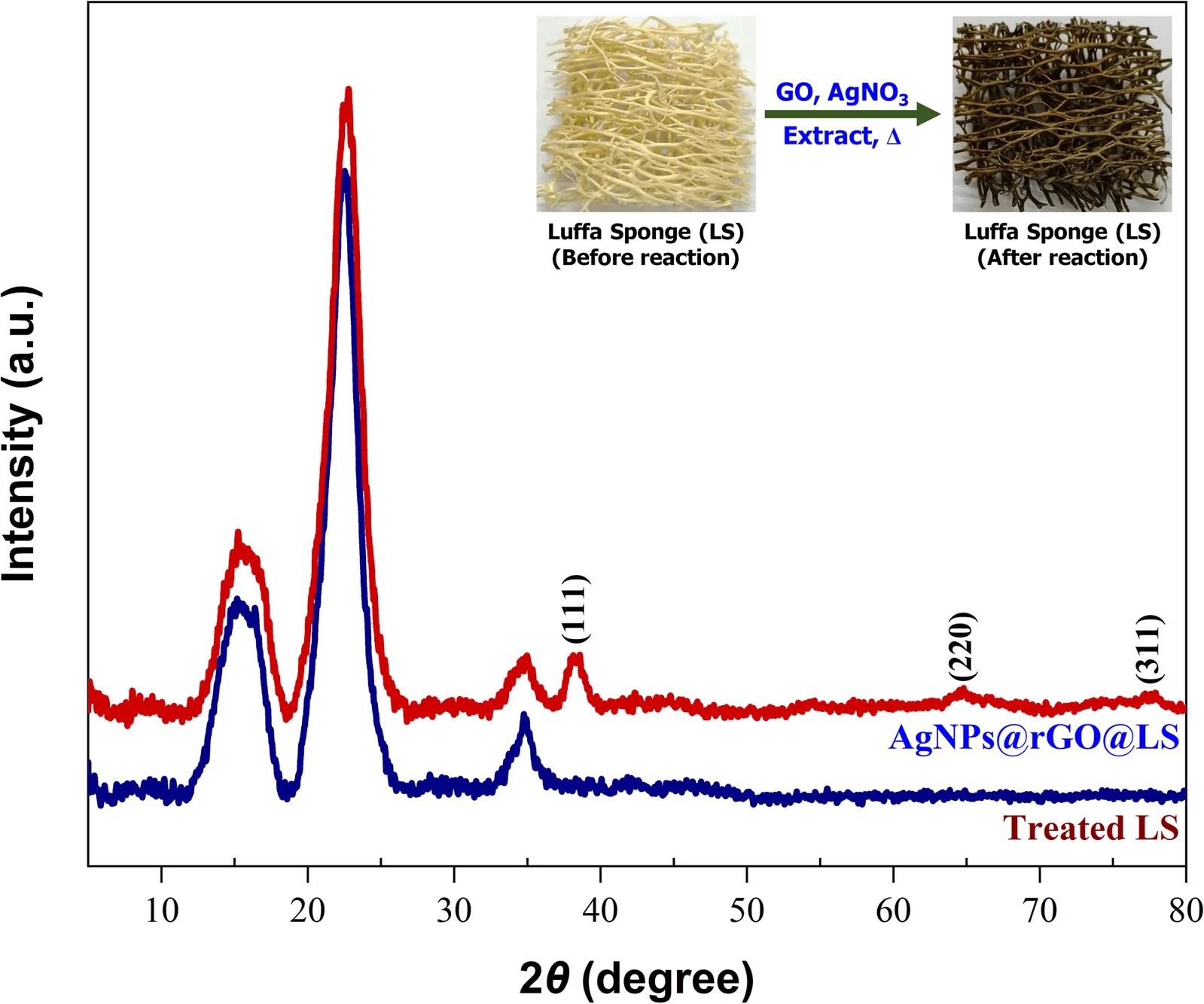
XRD patterns of treated LS and Ag/rGO@LS hybrid synthesized through an *in situ* approach using *P. chaba* stem extract.

To enhance the mechanical stability and prevent aggregation and nanoparticle leaching, the Ag/rGO composite was immobilized onto a luffa sponge (LS) scaffold. This was achieved through a facile *in situ* approach analogous to the synthesis of AgNPs@rGO, resulting in the successful formation of the ternary Ag/rGO@LS hybrid. The transition was macroscopically evidenced by a distinct color change of the LS from light brown to black (inset of Fig. 10), indicating the uniform deposition of the nanocomposite onto the natural framework. The utilization of the natural LS as a porous, three-dimensional support not only facilitates catalyst recovery but also mitigates the aggregation of AgNPs and Ag/rGO, thereby maintaining a high surface-area-to-volume ratio for improved catalytic performance.^26^ The XRD patterns provide definitive evidence for the successful integration of the Ag/rGO nanocomposite into the LS framework, as shown in Fig. 10. The diffractogram of the raw LS exhibits characteristic peaks at 2*θ* values of approximately 15°, 22°, and 34°, corresponding to the (110), (200), and (004) planes of crystalline cellulose I, respectively.^38,68^ Upon the *in situ* synthesis of the Ag/rGO@LS ternary hybrid, these primary cellulosic peaks remain prominent, confirming that the structural integrity of the natural fiber scaffold is preserved during the modification process.^26^ Crucially, the appearance of additional distinct reflections at 38°, 64°, and 78° corresponds to the (111), (220), and (311) planes of fcc silver, matching the previously confirmed Ag/rGOphase. The absence of a distinct rGO peak is likely due to the dominance of the LS signals, further verifying the successful formation of the hybrid catalyst.

**Fig. 11:**
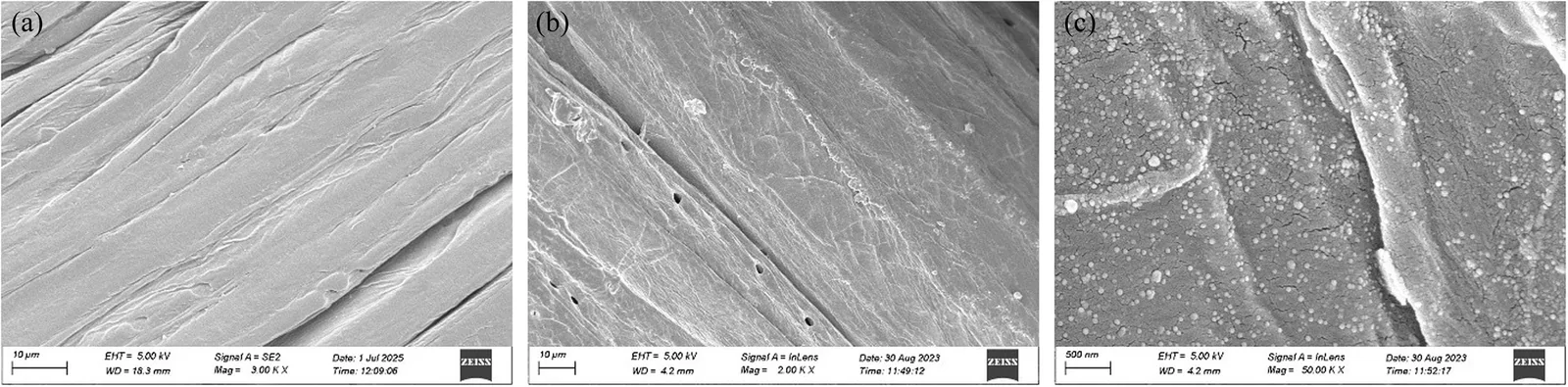
SEM images of (a) treated LS; (b) Ag/rGO@LS; and (c) high magnification view of (b).

The morphological evolution of the hybrid catalyst was investigated through SEM to verify successful immobilization. SEM image of the pristine LS reveal a characteristically smooth, macro-fibrous surface devoid of any particulate matter (Fig. 11a).^26,68^ Upon *in situ* synthesis, the Ag/rGO@LS composite undergoes a distinct morphological transformation: the porous LS scaffold becomes uniformly encapsulated by the wrinkled, ultrathin layers of the Ag/rGO composite. This hierarchical architecture is structurally divergent from both the AgNPs@LS and rGO@LS binary systems (SI, Fig. S8). FE-SEM reveals that the rGO sheets are anchored across the LS matrix, decorated with a dense population of spherical AgNPs. These nanoparticles exhibit a narrow size distribution, primarily ranging from 5 to 20 nm (Fig. 11b and c), suggesting a highly controlled nucleation process on the composite support. The high degree of dispersion suggests that the LS fiber effectively act as a stabilizing template, preventing Ag/rGO agglomeration.^26^ This hierarchical structure confirms the successful integration of the Ag/rGO phase onto the natural solid support, providing a robust, high-surface-area environment conducive to efficient catalytic activity.^26^

### Catalytic activity and reusability study of AgNPs@rGO@LS

**Fig. 12:**
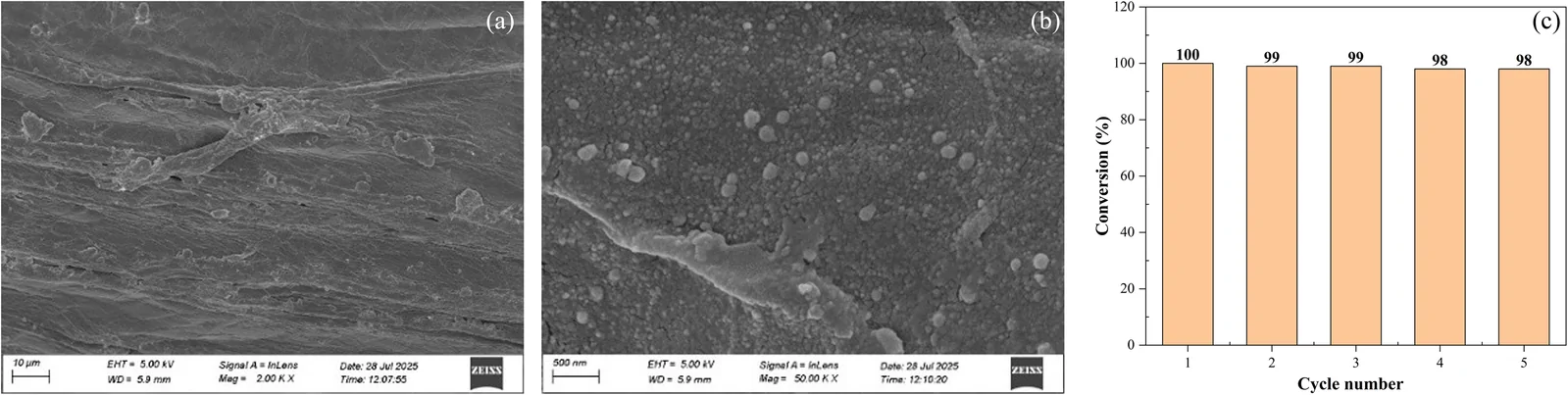
(a and b) FE-SEM images of Ag/rGO@LS hybrid after five consecutive catalytic cycle at different magnification and (c) recycle performance of the Ag/rGO@LS ternary nanohybrid catalyst for the 4-NP reduction.

The catalytic reusability and operational stability of the synthesized Ag/rGO@LS hybrid were evaluated over five consecutive reaction cycles for the reduction of 4-NP to 4-AP. Catalytic efficiency across all cycles was quantified by measuring the total percent conversion at a fixed 9 min reaction mark. The hybrid catalyst exhibited near-quantitative conversion (∼100%) in each cycle (Fig. 12c), demonstrating exceptional performance and durability. Control experiments using bare LS under identical reaction conditions demonstrated negligible 4-NP adsorption (less than 2%) and no catalytic reduction, confirming that the observed activity originates exclusively from the immobilized Ag/rGO nanocomposite. A comparative analysis of the catalytic reusability of AgNPs and Ag/rGO nanocomposites (Table 2) reveals that the Ag/rGO@LS composite, synthesized *via P. chaba* stem extract in this study, exhibits superior stability. This material maintained significantly higher catalytic efficiency over successive cycles compared to its counterparts during the reduction of 4-NP and various organic dyes, likely due to the synergistic effect between the AgNPs/rGO and the LS support.

**Table 2:** Comparative reusability of AgNPs and Ag/rGO nanocomposites for the catalytic reduction of 4-NP and various organic dyes^a^

Catalyst	Method of synthesis	Recycling performance			Ref.
		Model compound	Conversion (%)	No. of cycle	
rGO/AgNPs	Hydrothermal reduction	4-NP	∼84	5	66
		MB	∼95		
rGO/PDA/AgNPs	Dopamine-mediated synthesis	4-NP	90.2	5	25
		MB	79.3		
Cellulose/AgNPs	Cellulose-mediated synthesis	4-NP	100	3	69
AgNPs	*Ginkgo biloba* leaf-mediated synthesis	4-NP	∼85	5	70
		CR	∼85		
		MO	∼85		
		RhB	∼85		
AgNPs/rGO	NaBH_4_-mediated synthesis	4-NP	∼100	3	60
		MO	∼100		
AgNPs	*Mangifera indica* flower-mediated synthesis	4-NP	∼100	3	60
		MO	∼100		
AgNPs/rGO	Microwave irradiation	4-NP	83	5	64
Ag/rGO@LS	NaBH_4_-mediated synthesis	4-NP	95.46	5	26
Ag/rGO@LS	*Piper chaba* stem extract-mediated synthesis	4-NP	98	5	This work

a PDA = Polydopamine; CR = Congo red; RhB = rhodamine B.

Furthermore, FE-SEM analysis of the catalyst post-cycling revealed that the material maintained its structural integrity (Fig. 12), confirming that the morphological framework remains robust under reaction conditions. Partial distortion is observed after reusing SEM images regarding distribution of AgNPs, due to the consecutive effect of NaBH_4_.

## Conclusion

In summary, this study demonstrates a facile, benign, aqueous-phase immobilization of silver nanoparticle-decorated reduced graphene oxide (Ag/rGO) onto a luffa sponge (LS) scaffold. The Ag/rGO@LS ternary hybrid was synthesized *via* a one-step biogenic route using *P*. *chaba* stem extract, which served concurrently as a reducing and stabilizing agent. Under optimized conditions, spherical AgNPs (5–20 nm) were uniformly dispersed across crystalline rGO sheets and successfully anchored to the LS matrix. While the binary Ag/rGOnanocomposite exhibited significant initial catalytic activity for the reduction of 4-nitrophenol (4-NP) and the degradation of organic dyes (methyl orange and methylene blue), it suffered from poor reusability, with conversion efficiency dropping to 43% after five cycles. In contrast, the Ag/rGO@LS ternary composite displayed exceptional stability and near-quantitative reusability (∼100% conversion) at five consecutive cycles. Post-catalytic SEM analysis confirmed the structural integrity of the hybrid material, highlighting the advantages of integrating natural solid supports. However, key limitations remain: evaluation was restricted to batch-scale model solutions, lacking real wastewater complexity, and quantitative trace-metal leaching (ICP-MS) was not conducted. Future studies should focus on continuous-flow reactor systems, real industrial wastewater testing, and ICP-MS leaching analyses. Overall, this work highlights the potential of green chemistry for developing sustainable, high-performance supported nanomaterials for environmental remediation.

## Conflicts of interest

There are no conflicts to declare.

## Supplementary Material

RA-016-D6RA04267H-s001

## Data Availability

All data generated or analyzed during this study are included in this article. Backup data are available from the corresponding author(s) upon reasonable request. Supplementary information (SI): additional experimental details, characterization data, and mechanistic studies. See DOI: https://doi.org/10.1039/d6ra04267h.
