# Supplementary material for: Green synthesis of a recyclable Ag/rGO@LS nanocomposite: utilizing Piper chaba stem extract for the one-pot immobilization of Ag/rGO onto luffa sponge scaffolds
Source: RSC Adv. 2026 Aug 13;16(40):47267–79. doi: 10.1039/d6ra04267h (PMC13470673; doi:10.1039/d6ra04267h)
Supplement: Supplementary file 1 [file RA-016-D6RA04267H-s001.pdf]

Supplementary Information (SI) for RSC Advances.
This journal is © The Royal Society of Chemistry 2026

Supplementary Information

Green synthesis of a recyclable Ag/rGO@LS nanocomposite: Utilizing *Piper chaba* stem extract for the one-pot immobilization of Ag/rGO onto Luffa sponge scaffolds

Prianka Saha and Md. Mahiuddin*

Chemistry Discipline, Khulna University, Khulna-9208, Bangladesh

Treatment of Luffa sponge. Luffa (*Luffa aegyptica*) fibers were bought from a local market in Khulna, Bangladesh. The fibers were thoroughly washed with tap water followed by distilled water to eliminate adhering dirt and impurities. The cleaned samples were then oven-dried at 40 °C for 24 h. Subsequently, an alkali treatment was carried out using 5% sodium hydroxide solution with an appropriate amount of ethanol at 70 °C for 24 h to enhance the swelling ability of the cellulosic matrix.[1] After treatment, the fibers were rinsed repeatedly with distilled water until neutral pH was achieved and finally dried in an oven at 60 °C.

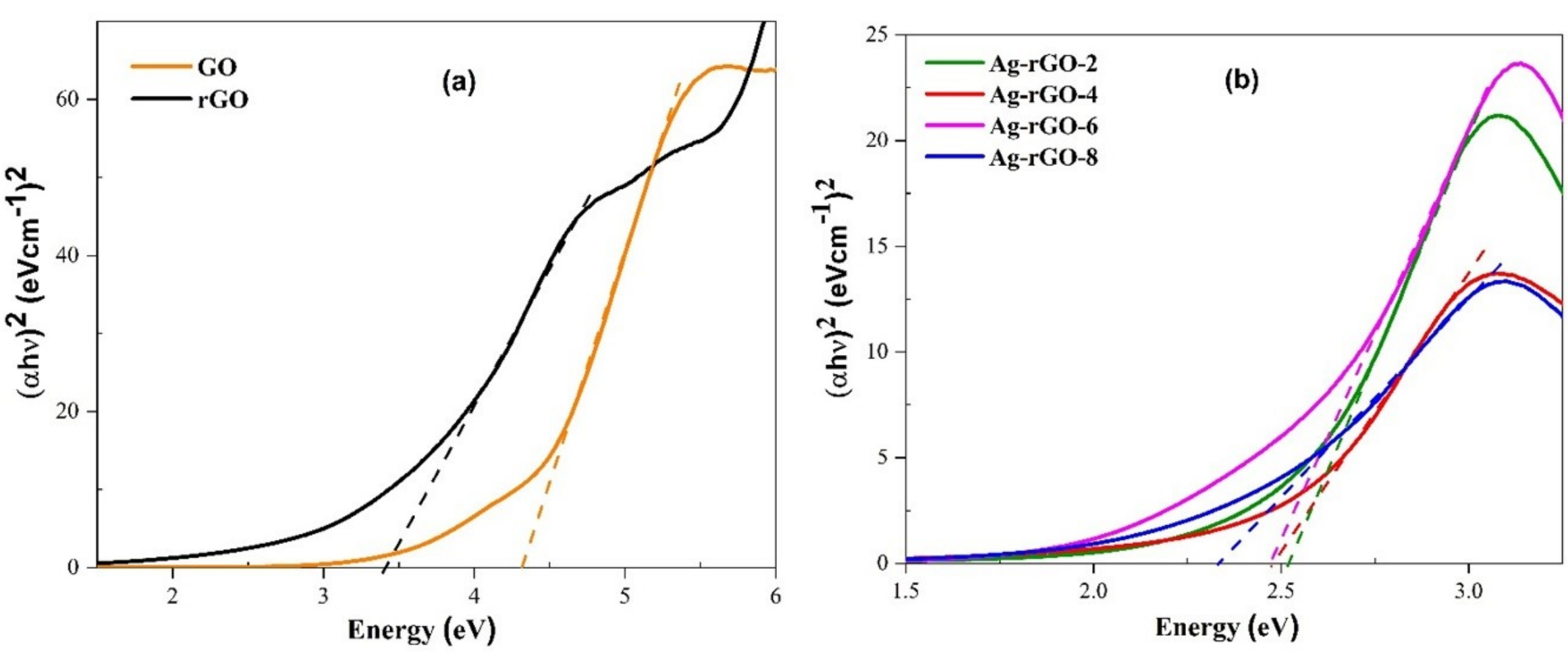

Figure S1. Tauc's plots[2] for determining the optical band gap of **(a)** GO and rGO and **(b)** Ag/rGO nanocomposites.

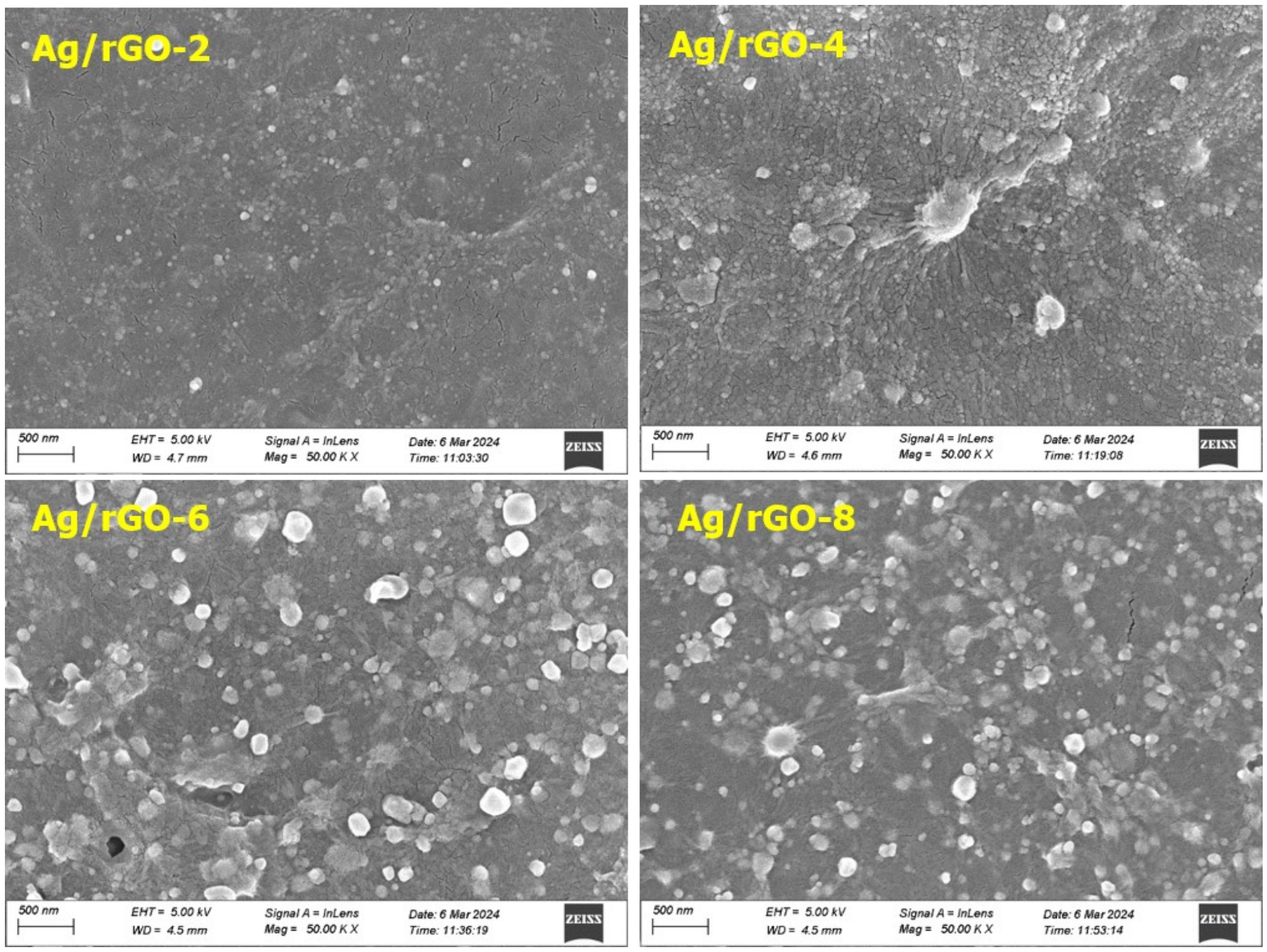

Figure S2. SEM images of Ag/rGO nanocomposites synthesized with different $AgNO_3$ concentration.

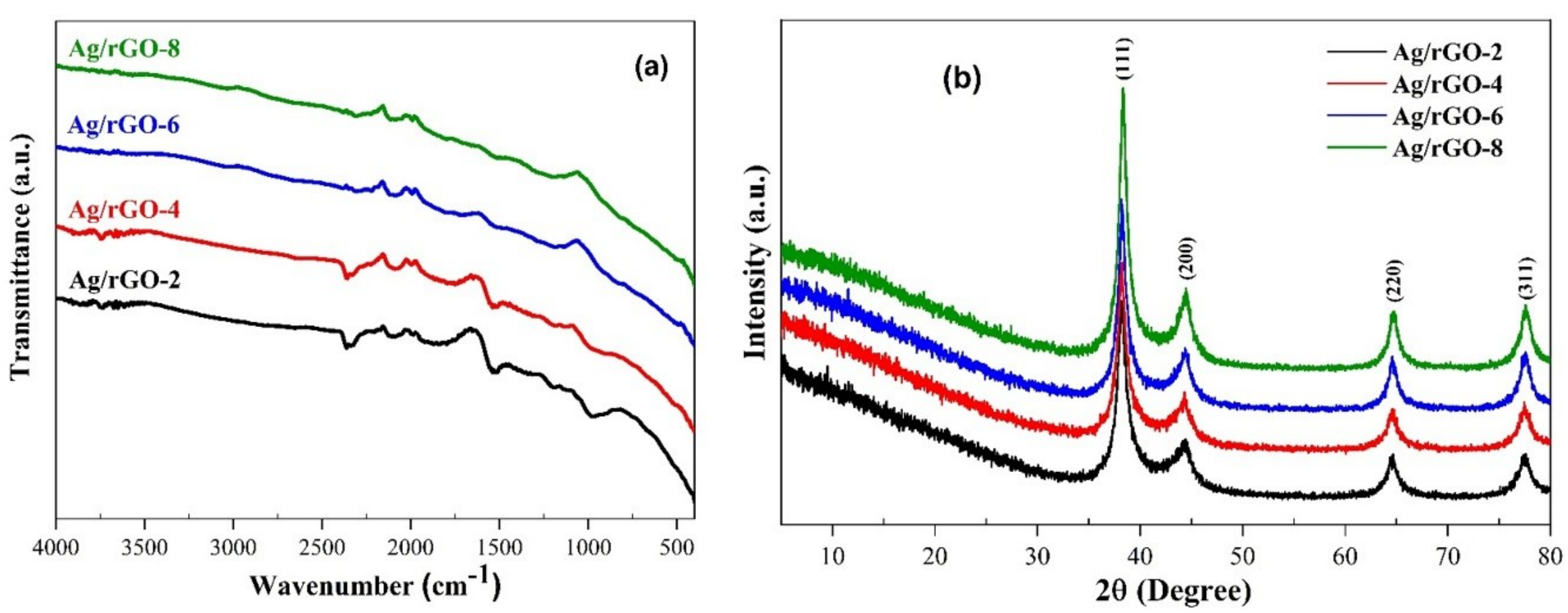

Figure S3. (a) FTIR spectra and **(b)** XRD patterns of Ag/rGO nanocomposites synthesized with different $AgNO_3$ concentration using *P. chaba* stem extract

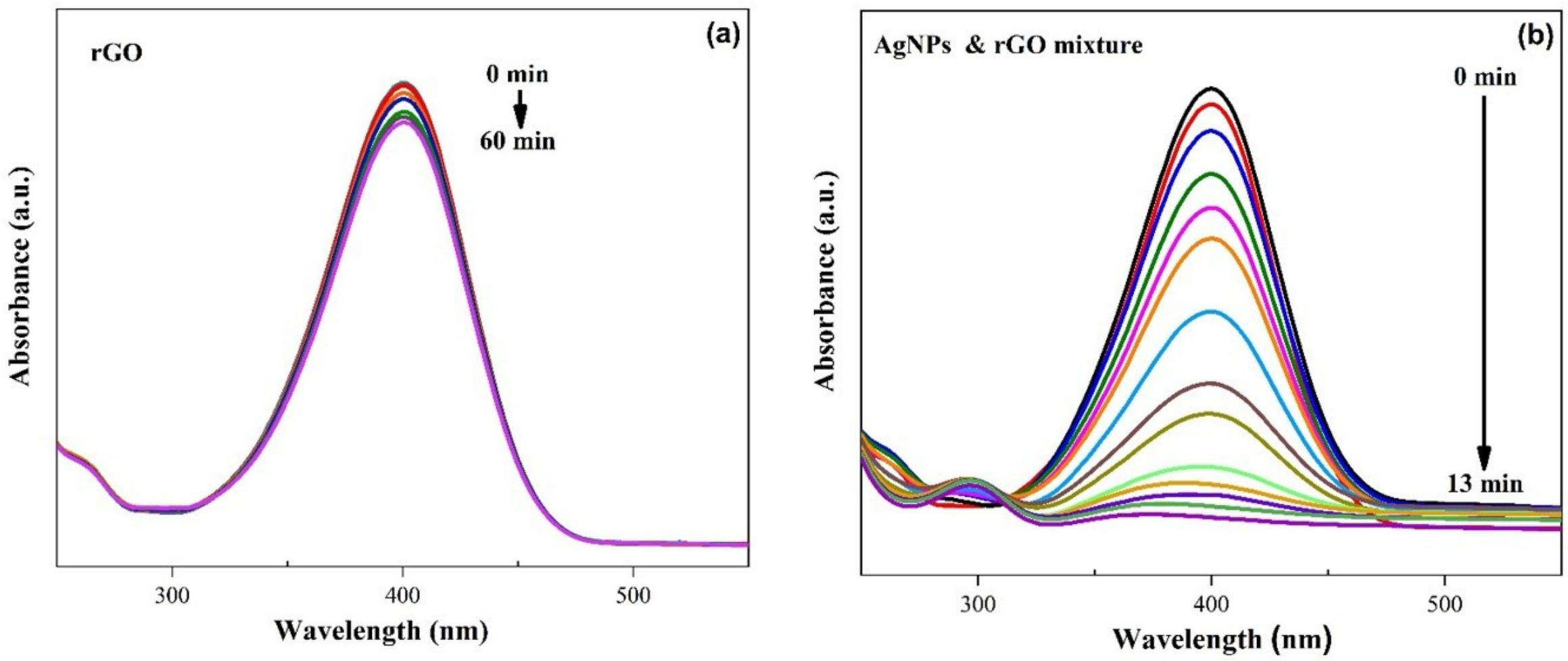

Figure S4. Time-dependence UV-vis spectra for the reduction of 4-NP to 4-AP by $NaBH_4$ in presence of *P. chaba* stem extract-mediated **(a)** rGO and **(b)** mixture of synthesized AgNPs & rGO as catalyst

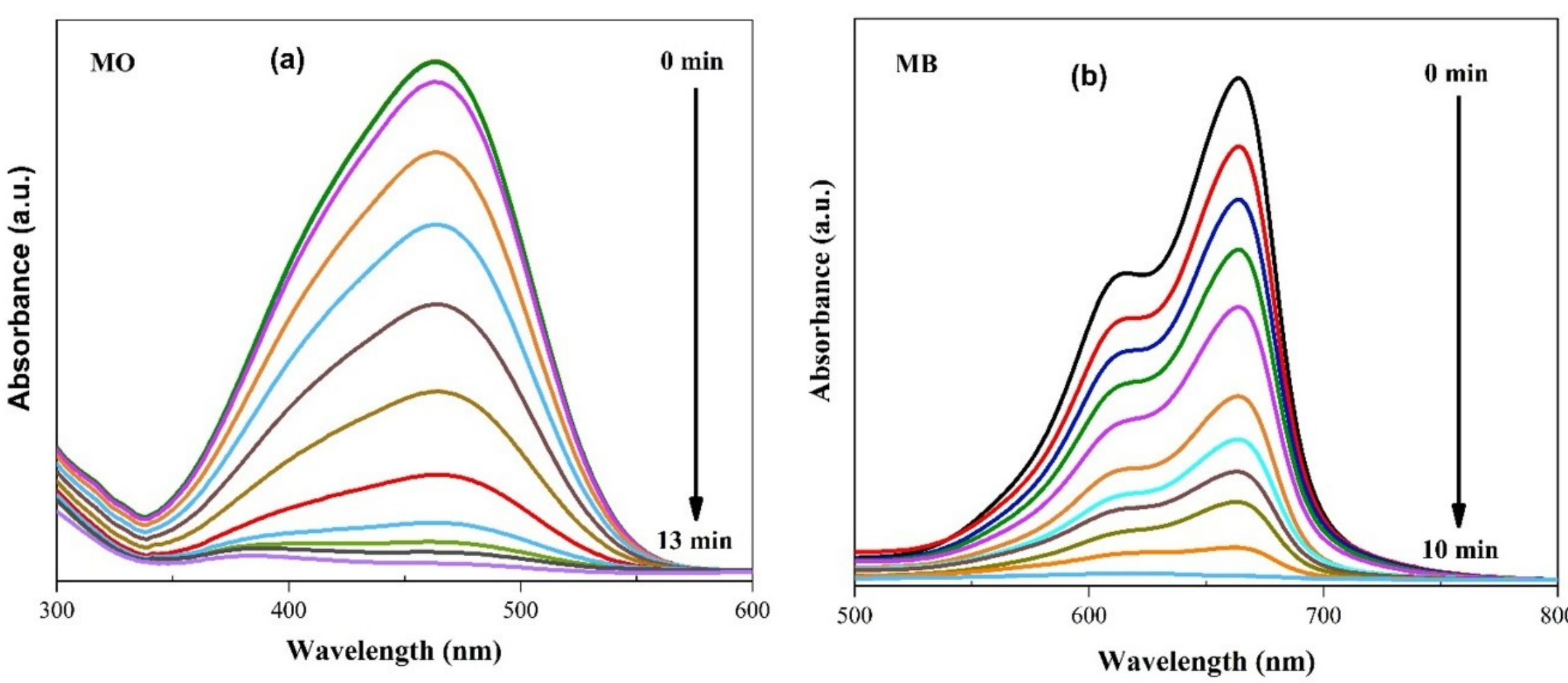

Figure S5. Time-dependence UV-vis spectra for the reduction of **(a)** MO and **(b)** MB by $NaBH_4$ in presence of Ag/rGO (AgNPs@rGO-4) nanocomposite as catalyst.

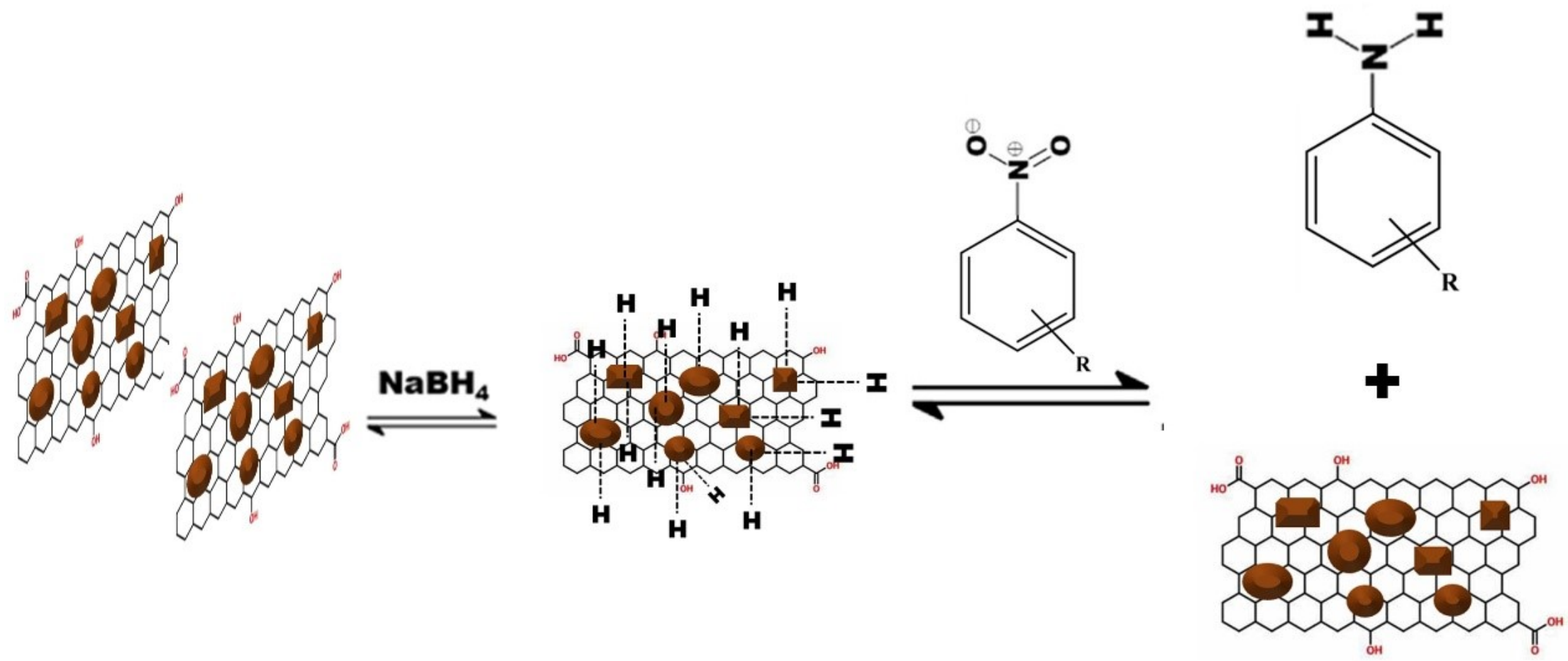

Figure S6. Possible catalytic mechanism of 4-nitrophenol reduction catalyzed by Ag/rGO nanocomposite[3,4]

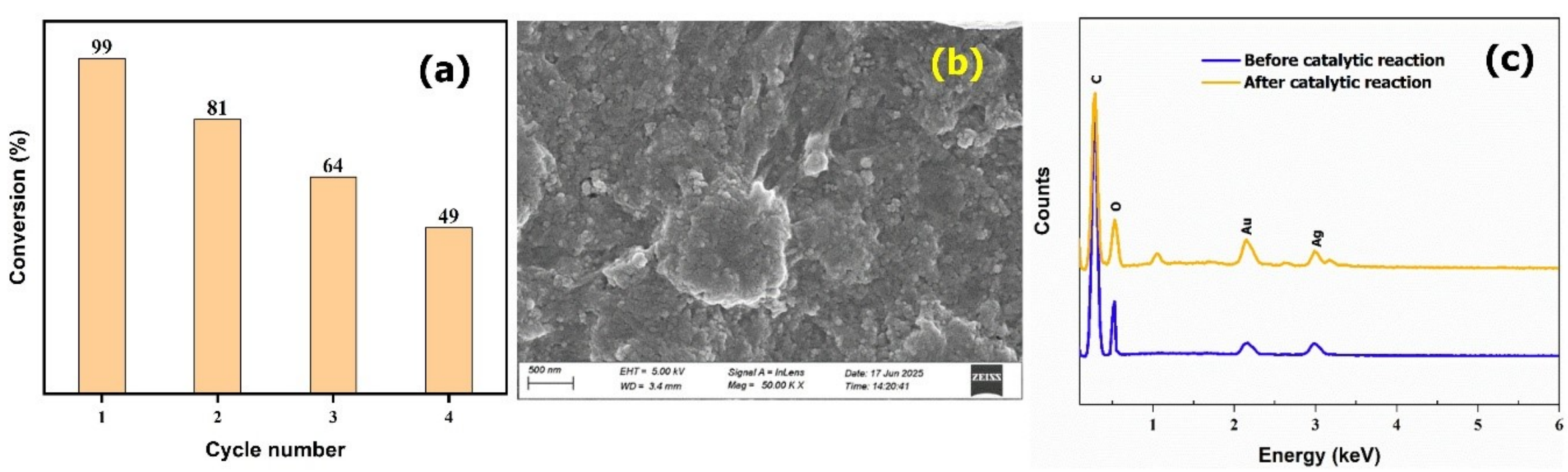

Figure S7. (a) Recycle performance of Ag/rGO nanocomposite as catalyst for the reduction of 4-NP to 4-AP; **(b)** SEM image and **(c)** EDX spectrum of Ag/rGOafter 2nd catalytic cycle.

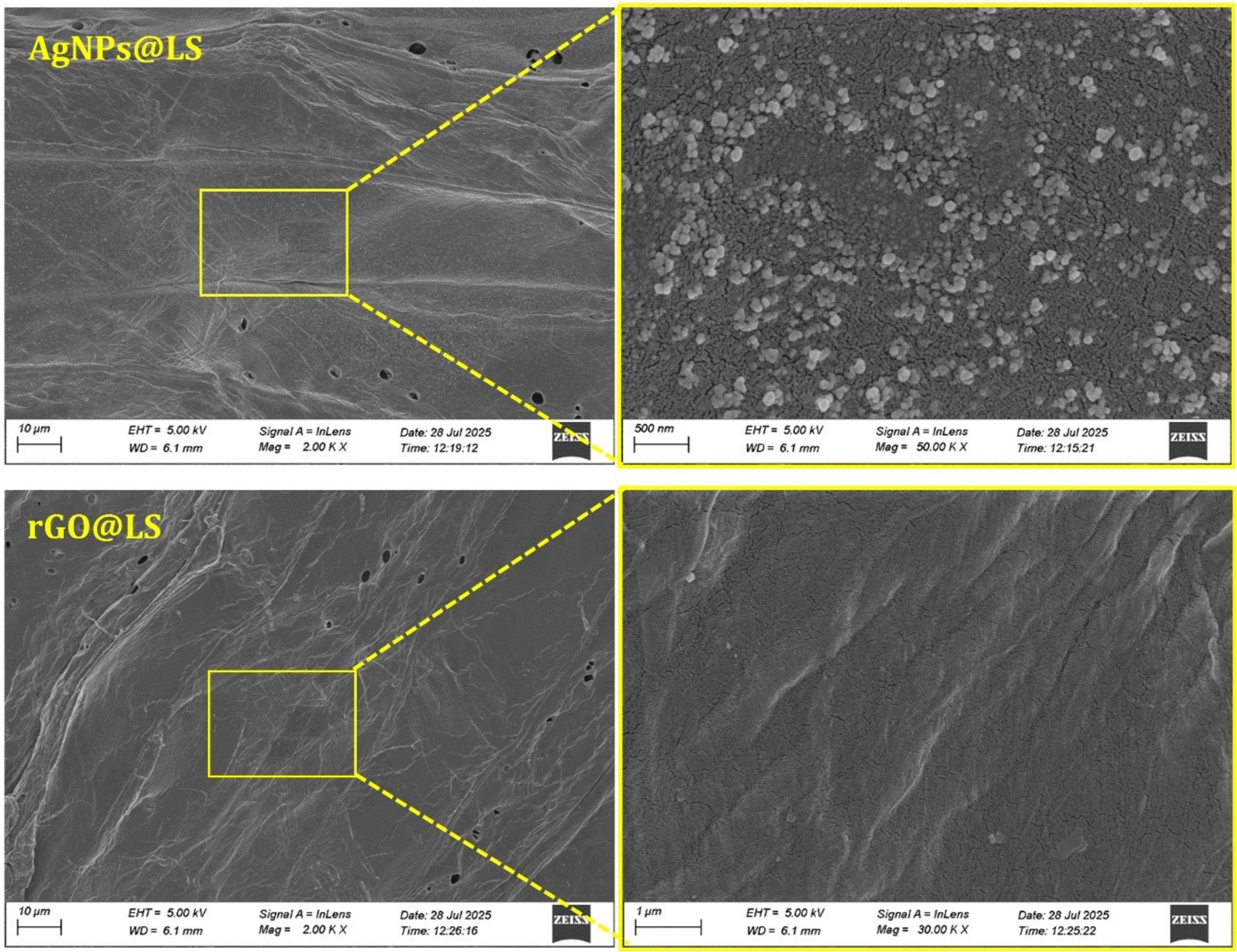

Figure S8. SEM images of AgNPs@LS and rGO@LS synthesized through one-pot approach using *P.chaba* stem extract

References

1 Y. Y. Liu, Y. H. Zhao, Y. Zhou, X. L. Guo, Z. T. Chen, W. J. Zhang, Y. Zhang, J. Chen, Z. M. Wang, L. T. Sun and T. Zhang, *Nanotechnology*, 2018, **29**, 315702.

2 J. Tauc, R. Grigorovici and A. Vancu, *Phys. status solidi*, 1966, **15**, 627–637.

3 X. Zhang, L. Chen, Y. Liu and Q. Duan, *Catalysts*, 2021, **11**, 1336.

4 M. Riaz, U. Sharafat, N. Zahid, M. Ismail, J. Park, B. Ahmad, N. Rashid, M. Fahim, M. Imran and A. Tabassum, *ACS Omega*, 2022, **7**, 14723–14734.
